# Regulating lactate-related immunometabolism and EMT reversal for colorectal cancer liver metastases using shikonin targeted delivery

**DOI:** 10.1186/s13046-023-02688-z

**Published:** 2023-05-10

**Authors:** Li Long, Wei Xiong, Fenwang Lin, Jiazhen Hou, Guihua Chen, Taoxing Peng, Yihao He, Rui Wang, Qin Xu, Yongzhuo Huang

**Affiliations:** 1 Artemisinin Research Center, Guangzhou University of Chinese Medicine, Guangzhou, 510450 China; 2 State Key Laboratory of Drug Research, Shanghai Institute of Materia Medica, Chinese Academy of Sciences, Shanghai, 201203 China; 3 Zhongshan Institute for Drug Discovery, Shanghai Institute of Materia Medica, Chinese Academy of Sciences, Zhongshan, 528437 China; 4 Department of Kidney Transplantation, Beijing Tsinghua Changgung Hospital, School of Clinical Medicine, Tsinghua University, Beijing, China; 5 School of Chinese Materia Medica, Nanjing University of Chinese Medicine, Nanjing, 210023 China; 6 NMPA Key Laboratory for Quality Research and Evaluation of Pharmaceutical Excipients, Shanghai, 201203 China

**Keywords:** Shikonin, Colorectal cancer liver metastasis, Glycolysis, Pyruvate kinase M2 (PKM2), Epithelial-mesenchymal transition (EMT), Immune microenvironment

## Abstract

**Background:**

There are few effective medications for treating colorectal cancer and liver metastases (CRLM). The interactions among glycolysis, epithelial-mesenchymal transition (EMT), and immune microenvironment contribute to the progression of CRLM. A main glycolytic enzyme pyruvate Kinase M2 (PKM2) is highly expressed in colorectal cancer and CRLM, and thus can be a potential therapeutic target.

**Methods:**

A therapeutic strategy was proposed and the shikonin-loaded and hyaluronic acid-modified MPDA nanoparticles (SHK@HA-MPDA) were designed for CRLM therapy via PKM2 inhibition for immunometabolic reprogramming. The treatment efficacy was evaluated in various murine models with liver metastasis of colorectal tumor.

**Results:**

SHK@HA-MPDA achieved tumor-targeted delivery via hyaluronic acid-mediated binding with the tumor-associated CD44, and efficiently arrested colorectal tumor growth. The inhibition of PKM2 by SHK@HA-MPDA led to the remodeling of the tumor immune microenvironment and reversing EMT by lactate abatement and the suppression of TGFβ signaling; the amount of cytotoxic effector CD8^+^ T cells was increased while the immunosuppressive MDSCs decreased.

**Conclusion:**

The work provided a promising targeted delivery strategy for CRLM treatment by regulating glycolysis, EMT, and anticancer immunity.

**Graphic abstract:**

An immunometabolic strategy for treating colorectal cancer liver metastases using the shikonin-loaded, hyaluronic acid-modified mesoporous polydopamine nanoparticles (SHK@HA-MPDA) via glycolysis inhibition, anticancer immunity activation, and EMT reversal. SHK@HA-MPDA can inhibit cytoplasmic PKM2 and glycolysis of the tumor and reduce lactate flux, and then activate the DCs and remodel the tumor immune microenvironment. The reduced lactate flux can reduce MDSC migration and suppress EMT.

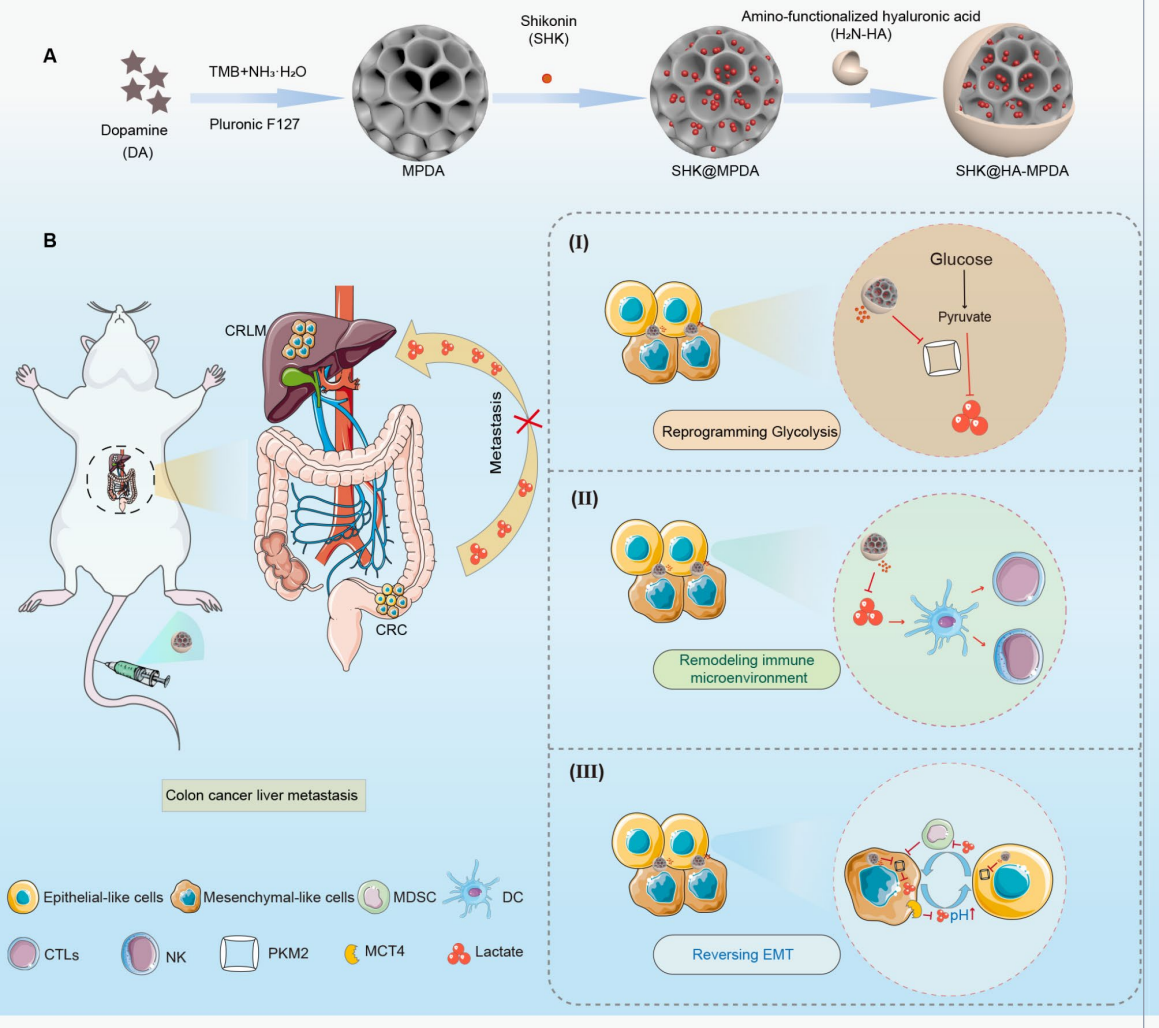

**Supplementary Information:**

The online version contains supplementary material available at 10.1186/s13046-023-02688-z.

## Introduction

Colorectal cancer (CRC) ranks third in terms of incidence, but second in terms of mortality, with more than new 1.9 million CRC cases and 935,000 deaths in 2020 [1]. Approximately 25 to 30% of colorectal patients develop liver metastases (CRLM) during the disease, and about 80% of CRLM patients have liver metastases that cannot be surgically removed at the time of initial diagnosis [2, 3]. The CRLM patients treated with palliative chemotherapy had a 1- and 5-year survival of 58.1% and 2.2%, respectively, while those receiving the best supportive care had a 1-year survival of 8.2% and no 5-year survivors [4]. Therefore, it is a pressing need to develop effective therapies for CRLM.

Aggravated aerobic glycolysis is a hallmark of cancer, aka the Warburg effect [5]. Pyruvate Kinase (PK) is the final rate-limiting step of glycolysis, which generates ATP and the end product lactate [6, 7]. A high level of PKM2 isoform has been observed in many tumors [8]. The upregulation of PKM2 was a correlative of later stage and lymph metastasis of CRC [9]. The highly active tetramer PKM2 allows the increased glycolytic flux to meet the biosynthetic demand for the rapid proliferation of cancer cells, while dimeric PKM2 is prevalent in the nucleus functioning as a protein kinase when cells were in the proliferative stage [10, 11]. Thus, blocking PKM2 can reduce lactate and ATP production and suppress PKM2 nucleus translocation, which is a potential strategy for anticancer therapy.

Notably, lactate from glycolysis was once considered a metabolic waste; nevertheless, it severs as an essential mediator bridging tumor metabolism and immune responses, and it shapes the immune cell fate and functions [12]. The increased intratumor lactate induced the differentiation of M2 macrophage and regulatory T cells (Tregs), suppressed dendritic cells (DCs), and blunted cytotoxic T cell and NK cell functions [13–16]. Therefore, lactate is a promising immunometabolic target for cancer therapy.

Furthermore, intensive aerobic glycolysis is closely associated with mesenchymal-like cells, in which the key enzymes including PKM2 are upregulated to facilitate the glycolytic flux [17, 18]. Epithelial-to-mesenchymal transition (EMT) is characterized by decreased intercellular adhesion and increased cell motility, and causes tumor metastasis [19, 20]. EMT and metabolic reprogramming are intertwined [21]. As a case in point, a high level of intratumor lactate can induce Snail and EMT by acidifying the tumor microenvironment and activating the TGF-β pathway [22]. TGF-β is a well-documented EMT inducer and can promote cancer cell migration and invasion along with upregulating EGFR expression [23, 24]. EGFR activation in turn facilitates nucleus PKM2 phosphorylation, which contributes to the expression of cyclin D1 and c-Myc, as well as tumor cell proliferation [10, 24]. The crosstalk of PKM2 with TGFβ–induced factor homeobox 2 (TGIF2) results in E-cadherin downregulation and initiation of EMT [25].

Herein, we proposed that tetramer PKM2 inhibition could reduce ATP and lactate production, thereby remodeling the tumor immune microenvironment, as well as deactivating TGF-β pathway and blocking the nuclear ectopic of PKM2 to reverse EMT.

Shikonin is a classic PKM2 inhibitor, which is a natural naphthoquinone compound isolated from *L.erythrorhizon*. In this study, the hyaluronic acid (HA)-modified mesoporous polydopamine (MPDA) nanosystem with shikonin encapsulation (SHK@HA-MPDA) was developed for a “one-stone-three-birds” therapeutic strategy to simultaneously regulate glycolysis, tumor immunity, and EMT. Due to the facile self-polymerization, MPDA can serve as a simple, rapid, and economical nanoparticle platform with many benefits such as high porosity, biocompatibility, ease to surface modification, and readiness for drug loading via π–π stacking and/or hydrogen bond interactions [26, 27]. Moreover, the surface-coating HA can serve as a targeted ligand for specifically binding with the overexpressed CD44 in the tumors for achieving tumor-targeting delivery.

## Results

### Overexpressed PKM2 in colorectal cancer influenced disease-specific survival

**Fig. 1:**
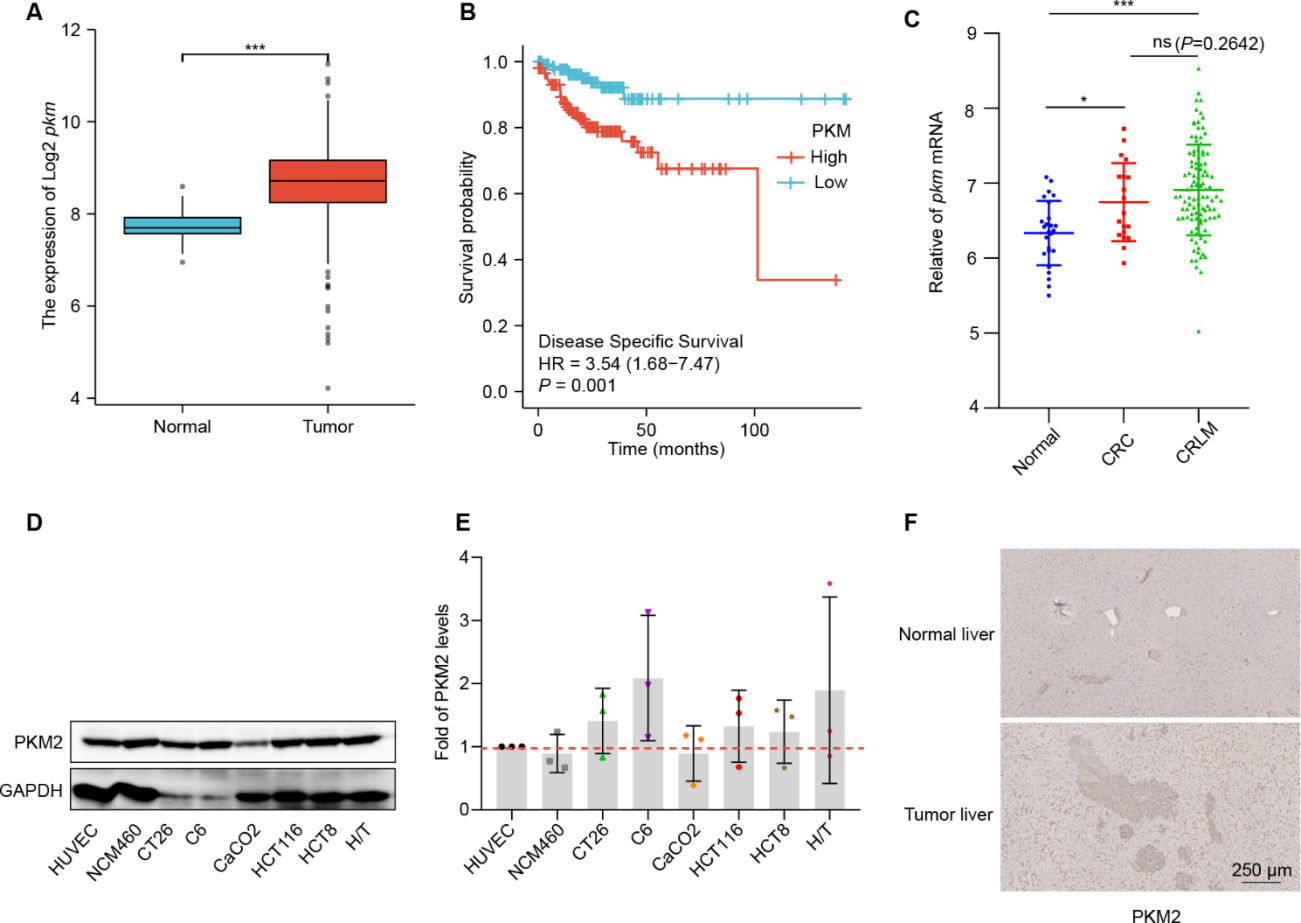
The upregulated expression of PKM2 in CRC and CRLM. (**A**) Primary CRC patients overexpressed *pkm2* mRNA compared to normal tissue in the TCGA database. (**B**) Patients with low *pkm2* expression in the TCGA database had significantly longer DSS (p = 0.01) than those with high *pkm2* expression. (**C**) In the GSE21510 data set, the expression of *pkm2* mRNA of liver tissue of normal, CRC, and CRLM. (**D**) and (**E**) The expression and grayscale analysis of PKM2 in various colorectal cancer cells. (**F**) Immunohistochemistry of PKM2 of liver tissue in normal or CRLM mice. CRC: Colorectal cancer cells; CRLM: Colorectal cancer liver metastasis. Data are presented as mean ± SD. *P < 0.05, **P < 0.01, ***P < 0.001

To investigate the role of PKM2 in CRC and CRLM, we performed a bioinformatics analysis based on the Cancer Genome Atlas (TCGA) database (https://portal.gdc.cancer.gov/) and GEO database (https://www.ncbi.nlm.nih.gov/geo/). The result showed the *pkm2* expression levels in CRC tissues were significantly higher than in the normal colonic mucosa (Fig. 1A), and disease-specific survival (DSS) of CRC with a low level of *pkm2* was superior to that with a high level (Fig. 1B). Furthermore, gene differential expression analysis in the GSE21510 cohort from the GEO database revealed that *pkm2* is also overexpressed in the tumor tissues of CRLM and CRC compared to the normal tissues (Fig. 1C). The protein level of PKM2 of colorectal cancer cells such as CT26, CaCO2, HCT8, HCT116, and resistant strains of HCT8 (H/T) were elevated compared to the normal cells like HUVEC and NCM460 (Fig. 1D and E). Besides, similar results were found in the CRLM mouse model (Fig. 1F). Therefore, PKM2 could serve as an essential participant in the progression of CRC and CRLM.

### Characterization of SHK@HA-MPDA nanoparticles

**Fig. 2:**
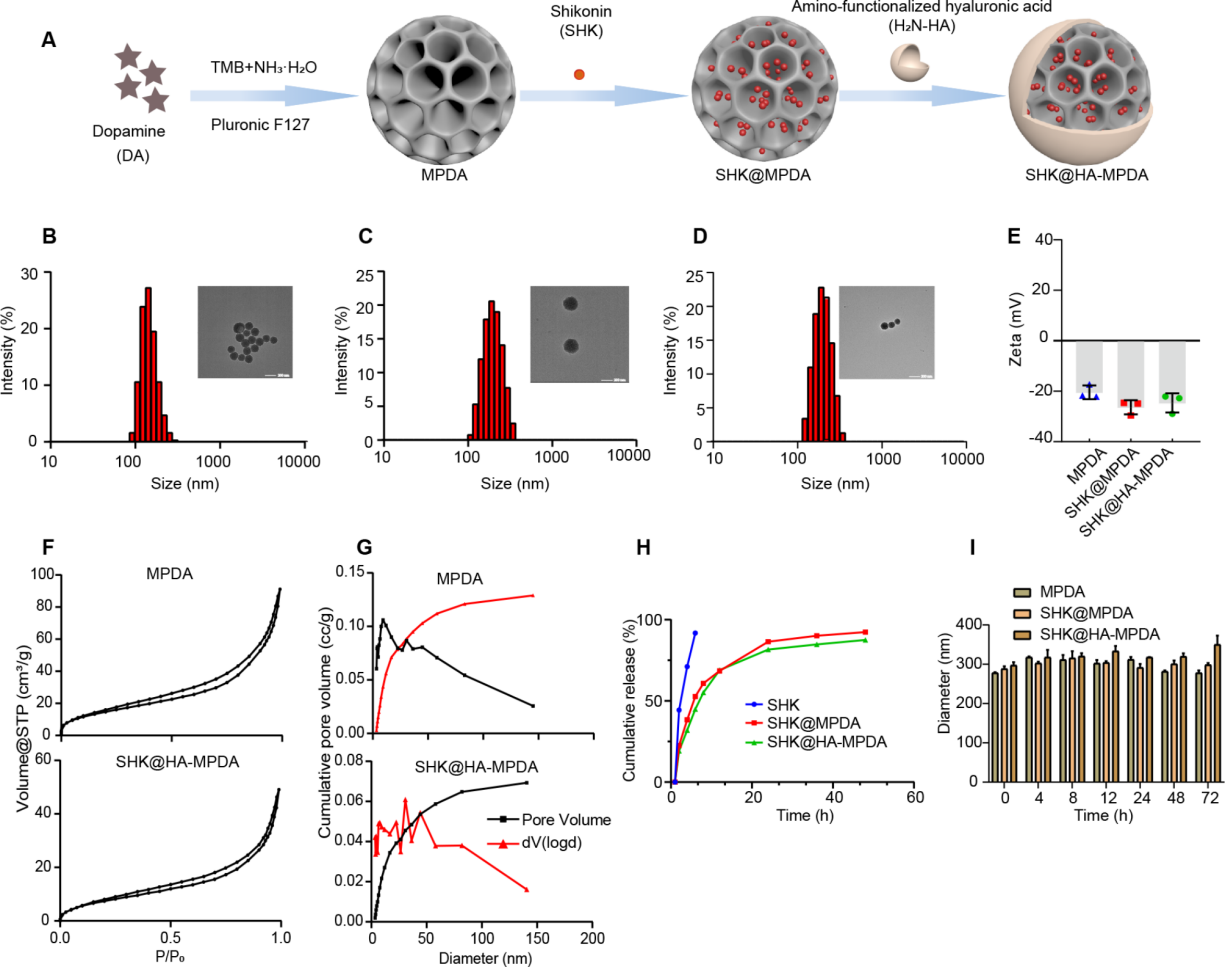
Characterization of nanoparticles (n = 3). (**A**) Schematic illustration of SHK@HA-MPDA nanoparticles. The particle size distribution and TEM of MPDA, SHK@MPDA, and SHK@HA-MPDA (**B**, **C**, and **D**). (**E**) The zeta potential of nanoparticles. (**F**) Nitrogen adsorption isotherms of nanoparticles. (**G**) The pore size distribution from the corresponding desorption isotherm. (**H**) The cumulative drug release of nanoparticles in PBS containing 0.5% SDS. (**I**) The serum stability of nanoparticles in PBS containing 10% FBS

SHK@HA-MPDA was synthesized based on the assembly of primary PDA and Pluronic F127 stabilized emulsion droplets. As shown in Fig. 2A, the TEM images of MPDA, SHK@MPDA, and SHK@HA-MPDA were spherical with uniform particle size distribution and good dispersion, and the mean size was measured to be 174.3 nm, 187.8 nm, and 189.1 nm respectively (Fig. 2B, C, and D). The nanoparticles showed negative ζ-potential (Fig. 2E). The Brunauer − Emmett − Teller (BET) surface areas were calculated to be 53.3 and 28.5 m^2^/g, and the corresponding pore sizes are 3.8 and 3.4 nm for MPDA and SHK@HA-MPDA, respectively (Fig. 2F and G). The encapsulation efficiency and drug-loading capacity of SHK in SHK@HA-MPDA were 62.8% and 3.6%, respectively. The results also revealed a sustained drug release profile (Fig. 2H) and good stability of the nanoparticles in a serum-containing medium (Fig. 2I).

**Fig. 3:**
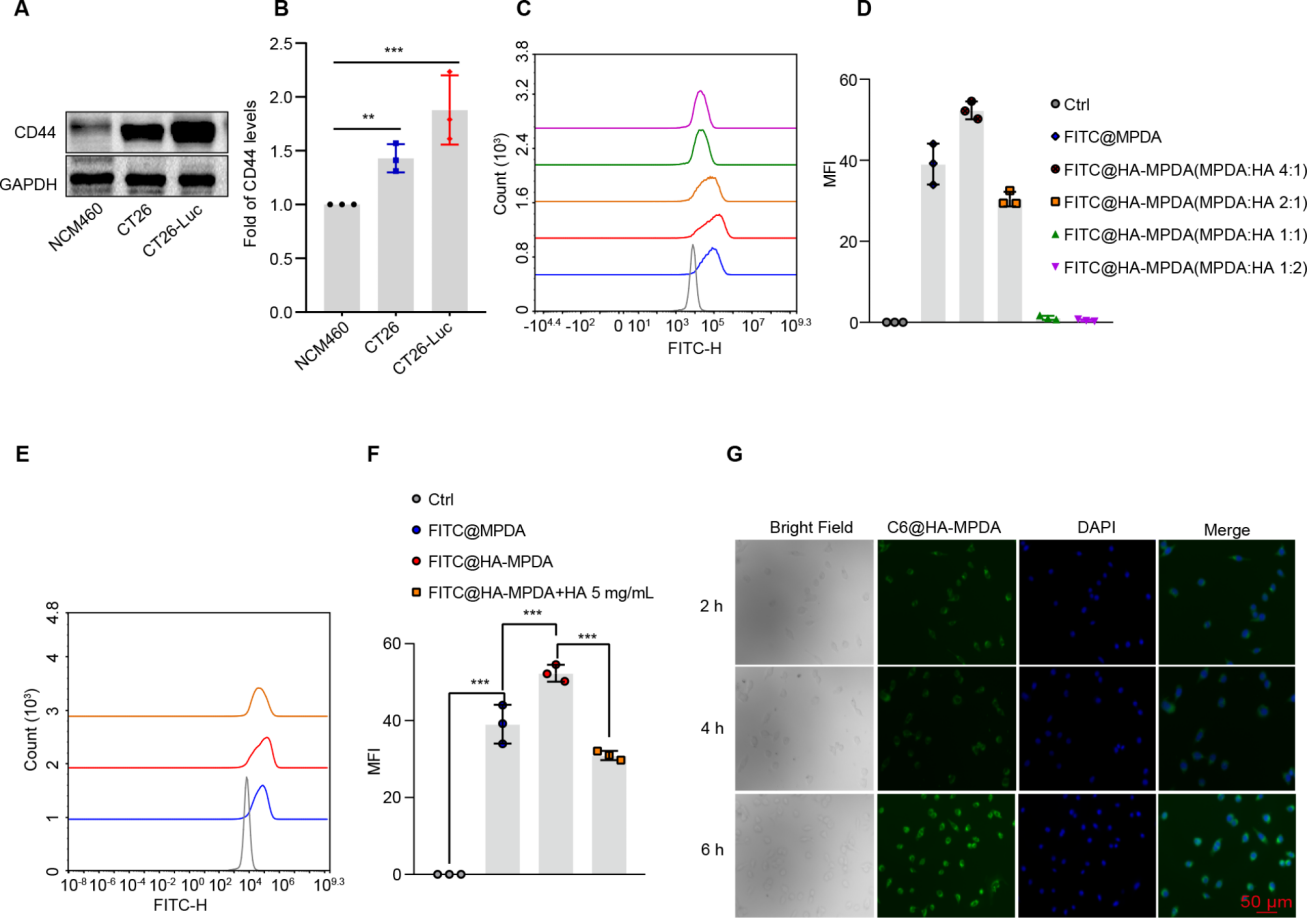
Cellular uptake of nanoparticles. (**A**) and (**B**) The expression and grayscale analysis of CD44 in NCM460, CT26, and CT26-Luc. (**C**) The cellular uptake of different ratios of HA and MPDA in CT26 cells and the statistical analysis of cellular efficiency in (**D**). (**E**) The cell uptake of nanoparticles with or without HA in CT26 cells and the statistical analysis of cellular efficiency in (**F**). (**G**) The inverted microscope image of uptake of C6@HA-MPDA along with time in CT26 cells. Data are presented as mean ± SD (n = 3). *P < 0.05, **P < 0.01, ***P < 0.001

CD44 is a membrane receptor for HA [28], and is an essential signaling molecule involved in tumor progression and metastasis, and the overexpressed CD44 is an unfavorable prognostic factor for CRC patients [29]. As shown in Fig. 3A and B, the CD44 protein was overexpressed on the colorectal cancer cells compared with the normal colon epithelial cells (NCM460). Therefore, the HA-coating MPDA nanoparticles were used as a targeted drug delivery carrier. The optimized modification ratio of MPDA and HA was 4:1, which showed the highest cellular uptake in the CT26 cells (Fig. 3C and D). Furthermore, the cellular uptake efficiency of the HA-MPDA was decreased when the cells were pretreated with free HA (Fig. 3E and F). It thus suggested that HA-mediated targeting delivery is a key mechanism. The uptake efficiency of HA-MPDA showed a time-dependent manner (Fig. 3G).

### Down-regulation of glycolytic lactate production in colorectal cancer cells

**Fig. 4:**
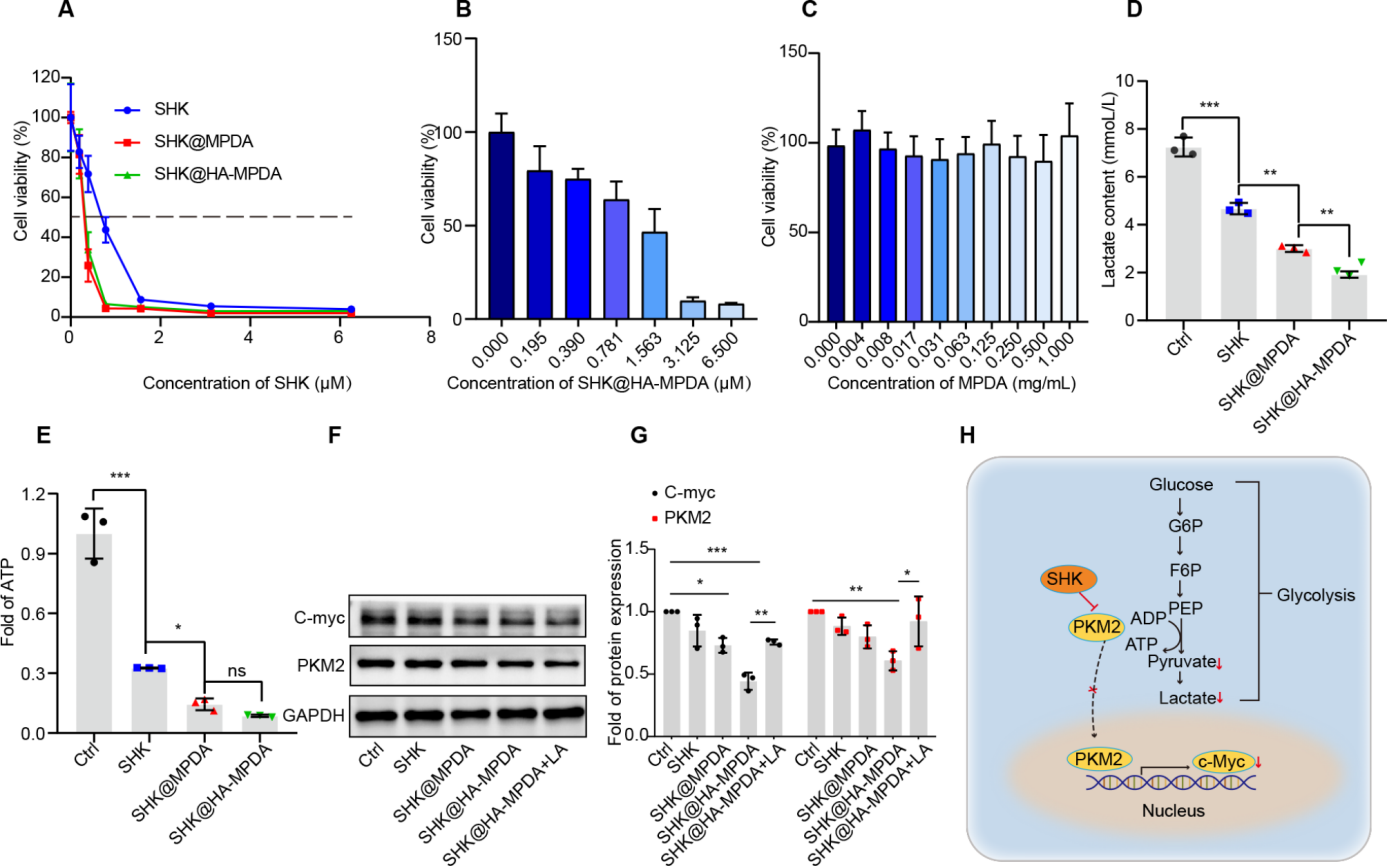
The regulation glycolysis and cell proliferation of nanoparticles in CT26 cells. (**A**) The cytotoxicity test of nanoparticles. (**B**) The cytotoxicity test of SHK@HA-MPDA in NCM460 cells. (**C**) The cytotoxicity test of MPDA in CT26 cells. (**D**) and (**E**) Quantitative of exocellular lactate and intracellular ATP of CT26 cells treated with nanoparticles. (**F**) and (**G**) The expression and grayscale analysis of PKM2 and c-Myc in CT26 cells after treatment by nanoparticles. (**H**) The Schematic mechanism of the nanoparticles regulating glycolysis and proliferation. Data are presented as mean ± SD (n = 3). *P < 0.05, **P < 0.01, ***P < 0.001

SHK is a glycolytic suppressor via PKM2 inhibition and thus can reduce lactate production. Lactate is not only an end product of glycolysis but also a signaling molecule that induces cancer metastasis, angiogenesis, and immunosuppression [30, 31]. The mycoplasma test in CT26 cells and NCM460 cells was negative (Fig. S1). The IC_50_ of SHK@HA-MPDA was 0.3 µM, which was about half of free SHK (Fig. 4A). The IC50 of SHK@HA-MPDA in NCM460 was 1.05 µM, exhibiting more than 3-fold higher than that of CT26 cells (Fig. 4B). It suggested that SHK@HA-MPDA showed preferential action on the tumor cells. The empty MPDA had little toxicity to CT26 cells (Fig. 4C). As shown in Fig. 4D and E, SHK significantly decreased the production of lactate and ATP of the CT26 cancer cells, because of PKM2 inhibition. SHK@HA-MPDA nanosystem substantially downregulated the expression of oncogenes c-Myc compared with the untreated group; the addition of exogenous lactate reversed this effect (Fig. 4F and G). It is known that native tetrameric PKM2 in the cancer cell cytoplasm is phosphorylated or acetylated, which can transfer to a dimeric/monomeric form that translocated into the nucleus; nuclear PKM2 causes oncogene transcription (e.g., c-Myc) [32], as illustrated in (Fig. 4H).

### Activating the dormant tumor immune microenvironment

**Fig. 5:**
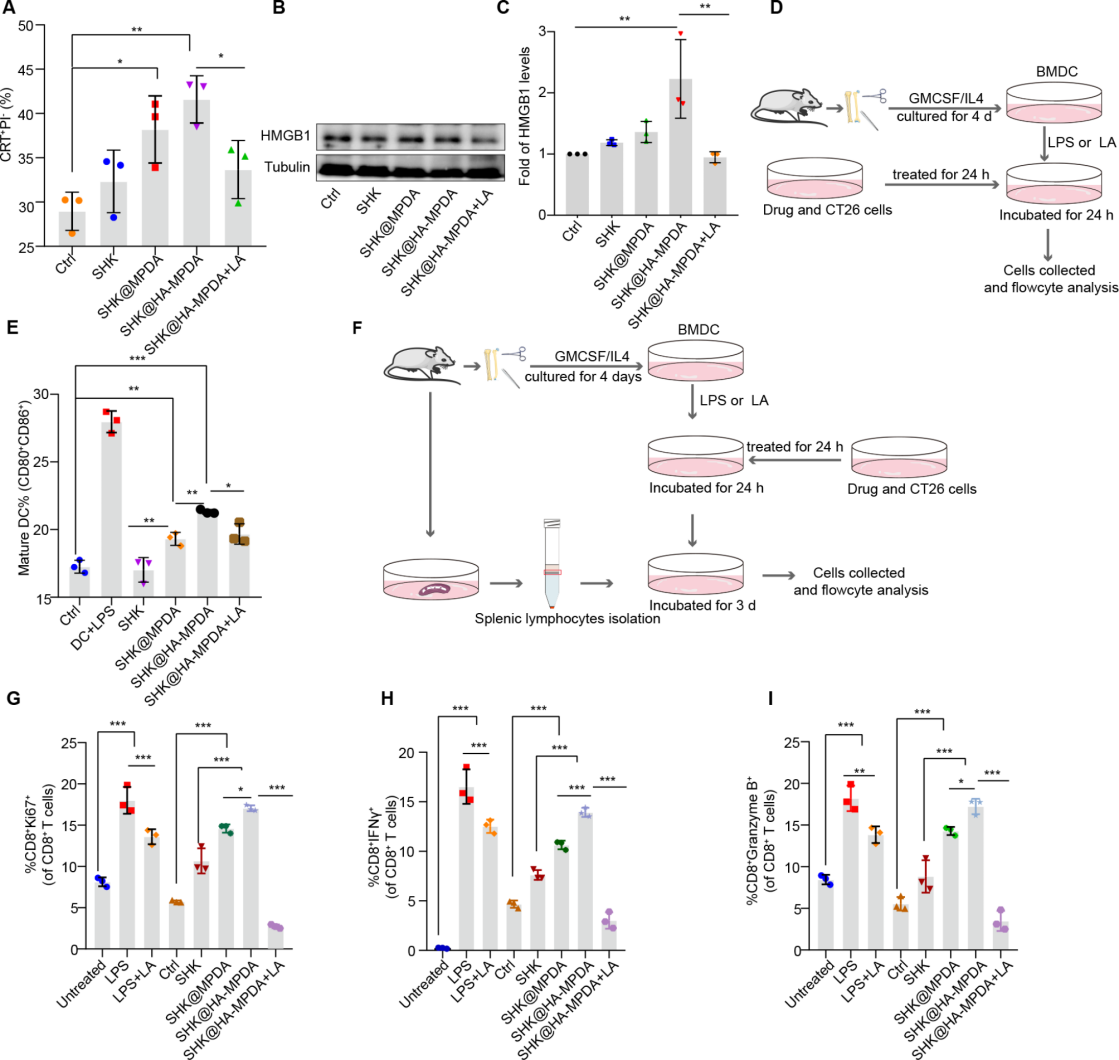
Remodeling of immunometabolism. (**A**) Analysis of calreticulin eversion in CT26 cells by flow cytometry. (**B**) and (**C**) Western blot analysis of the released HMGB-1 of CT26 cells and grayscale analysis of HMGB1. (**D**) Schematic of the ICD stimulated maturation of BMDC. (**E**) CD80^+^CD86^+^ DC subsets were stimulated by CT26 cells after treatment (LPS: 200 ng/mL; LA: lactate 5 mM). (**F**) Schematic of ICD activated T cell immunity. (**G–I**) The CD8^+^T subsets (CD8^+^Ki67^+^) and the cytotoxic substances of CD8^+^T (CD8^+^IFNγ^+^, CD8^+^Granzyme B^+^) (Untreated: CT26 co-incubated with DC and spleen lymphocytes; LPS: 200 ng/mL; LA: 10 mM). Data are presented as mean ± SD (n = 3). *P < 0.05, **P < 0.01, ***P < 0.001

SHK can induce immunogenic cell death (ICD) which triggers damage-associated molecular patterns (DAMPs) that are characterized by calreticulin (CRT) eversion and the release of high mobility group box 1 (HMGB-1) [33, 34]. SHK@HA-MPDA nanosystem induced CRT eversion and HMGB1 expression in the CT26 cells, but this process could be blocked by the addition of exogenous lactate (Fig. 5A, B, and C ). The results suggested that SHK@HA-MPDA was efficient to induce ICD and diminish lactate production, and thus would be favorable to augmenting the immune activation.

Dendritic cells (DCs) are the primary class of antigen-presenting cells for eliciting immunity. The activation efficiency of BMDCs with co-incubation of the drug-treated tumor cells was examined (Fig. 5D). Our results indicated that the tumor cells pretreated with SHK@HA-MPDA efficiently induced BMDC activation (Fig. 5E). The activated BMDCs were further evaluated for their effects on T cells by co-incubation with the splenic lymphocytes (Fig. 5F). It was revealed that CD8^+^ T cells, the major effector cytotoxic T lymphocytes (CTLs), was activated (Fig. 5G–I, and Fig. S3A). Notably, lactate can reduce the maturation of BMDCs and cytotoxic efficacy of CD8^+^ T cells (Fig. 5G, H, and I), further confirming the negative impact of lactate on anticancer immunity.

### EMT reversal by suppressing glycolysis and lactate production

The transcriptional repressor Snail can trigger EMT, which is defined by the loss of adhesion between cells accompanied by reduced expression of E-cadherin, and is responsible for tumor invasion and metastasis [35–37]. In comparison with the normal epithelial cells (NCM460), CT26 cells showed a much lower E-cadherin level (Fig. S2A and S2B), suggesting the EMT characteristic. SHK can reverse EMT, as evidenced by the downregulation of Snail and E-cadherin (Fig. S2C and S2D). In a colorectal cancer liver metastasis mouse model (Fig. S2E), SHK treatment resulted in the EMT reversal as shown as the upregulated E-cadherin and the downregulated vimentin and α-SMA (Fig S2F, S2G, S2H, S2I, and S2J). The data suggested SHK was a potential drug candidate for reversing EMT.

**Fig. 6:**
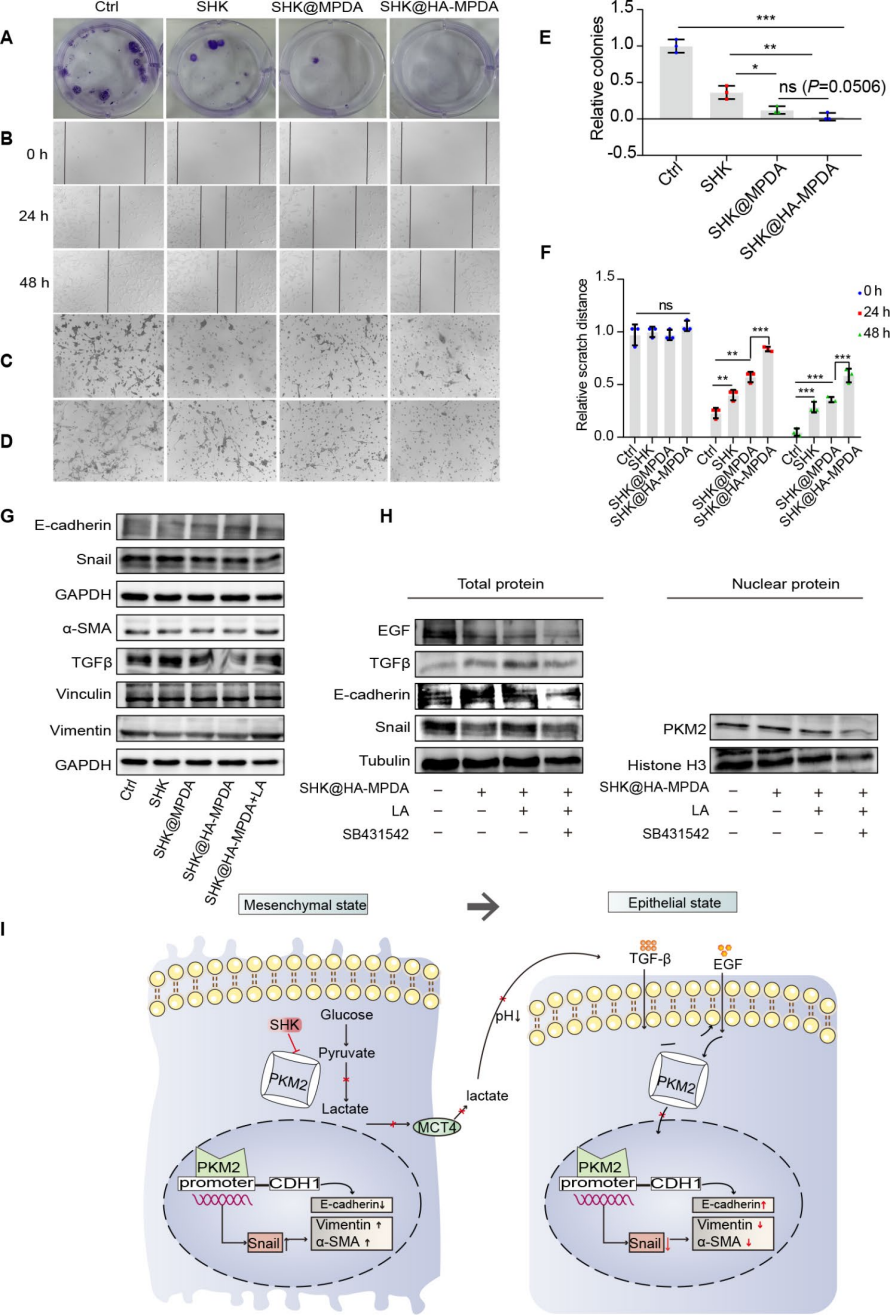
The crosstalk between EMT and glycolysis. (**A**) After treatment with nanoparticles, the representative images of colony-forming cells of CT26 cells and the statistical analysis of cell clusters in (**E**). (**B**) The representative images of scratch assay of CT26 cells and the statistical analysis of distance in (**F**). The representative images of migration (**C**) and invasion (**D**) of CT26 cells after suffering nanoparticles. (**G**) Expression levels of epithelial or mesenchymal markers in CT26 cells were analyzed by western blotting. (**H**) Expression levels of epithelial or mesenchymal markers of CT26 cells after suffering SHK@HA-MPDA but intervening with or without LA or SB431542 were analyzed by western blotting (LA: 10 mM; SB431542: 5 µM). (**I**) The schematic of the mechanism between EMT and glycolysis in mesenchymal-like cells and epithelial-like cells. Data are presented as mean ± SD (n = 3). *P < 0.05, **P < 0.01, ***P < 0.001

The plate cloning experiments revealed that SHK@HA-MPDA efficiently suppressed the CT26 cell proliferation (Fig. 6A and E). Furthermore, SHK@HA-MPDA dramatically reduced the migration of CT26 cells (Fig. 6B, F, and C) and retarded the invasion of CT26 cells (Fig. 6D). These results demonstrated that SHK@HA-MPDA could potentially apply for inhibiting tumor metastasis.

Lactate is the end product of glycolysis and induces TGFβ1 upregulation and the acidic microenvironment [38]. TGFβ1 is known as a potent EMT inducer, and TGFβ participates in dimer nuclear PKM2 formation and induces E-cadherin expression [25]. Therefore, the effect of SHK@HA-MPDA was investigated. Notably, CT26 cells were mesenchymal-like and characterized by the low expression of E-cadherin (Fig. S4A and S3B). Figure 6G and Fig. S2K demonstrate that SHK@HA-MPDA can reverse EMT (with the downregulated Snail and vimentin, and increased E-cadherin) and reduce the phosphorylated TGFβ level. However, the treatment effect can be revoked by adding exogenous lactate (10 mM) (Fig. 6G) or TGFβ receptor inhibitor (SB431542) (Fig. 6H and Fig. S2L). Therefore, the EMT reversal by SHK@HA-MPDA relied on the mechanisms of inhibiting tetramer PKM2-mediated glycolysis and reducing lactate-driving mesenchymal-like differentiation via blocking the TGFβ/EGF-cascaded PKM2 nuclear ectopic (Fig. 6I).

To further verify the critical role of lactate in EMT, we used another EMT model (TGFβ-induced SW480 cells). Different from the mesenchymal-like CT26 cells, SW480 cells were epithelial-like, which however can be induced EMT by giving TGFβ through activating TGFβ/EGF cascaded dimer PKM2 nuclear ectopic, but SB431542 can block the EMT process (Fig. S4C and S4D). As shown in Fig. S4E, the TGFβ-treated SW480 secreted more lactate than the untreated cells (epithelial-like), which suggested that the mesenchymal-like cancer cells increasingly relied on glycolysis. Furthermore, exogenous lactate can also activate the TGFβ/EGF and further induce Snail, E-cadherin, and PKM2 nuclear ectopic to induce EMT, but not influence the cytoplasmic tetramer PKM2 (Fig. S4F and S4G). However, lactate-induced EMT in the SW480 cells can be inhibited by pH neutralization or SB431542 treatment (Fig. S4F).

### In vivo investigation of intratumor drug accumulation

MPDA is an up-conversion material that can convert NIR light into heat and exhibits both concentration-dependent and power-dependent patterns (Fig. S5A, B, and C) and photothermal stability as well (Fig. S5D). Therefore, such a heat performance can be used for monitoring the intratumor accumulation of SHK@HA-MPDA. The results revealed that both SHK@MPDA and SHK@HA-MPDA showed maximal heat effect at 30 h post-injection, suggesting the peak of intratumor accumulation at the moment (Fig. S5E and F). The heat performance of SHK@HA-MPDA was better than SHK@MPDA, indicating the former had higher intratumor accumulation (Fig. S5G and H).

### Suppressing the migration of MDSCs in the CRC tumor

**Fig. 7:**
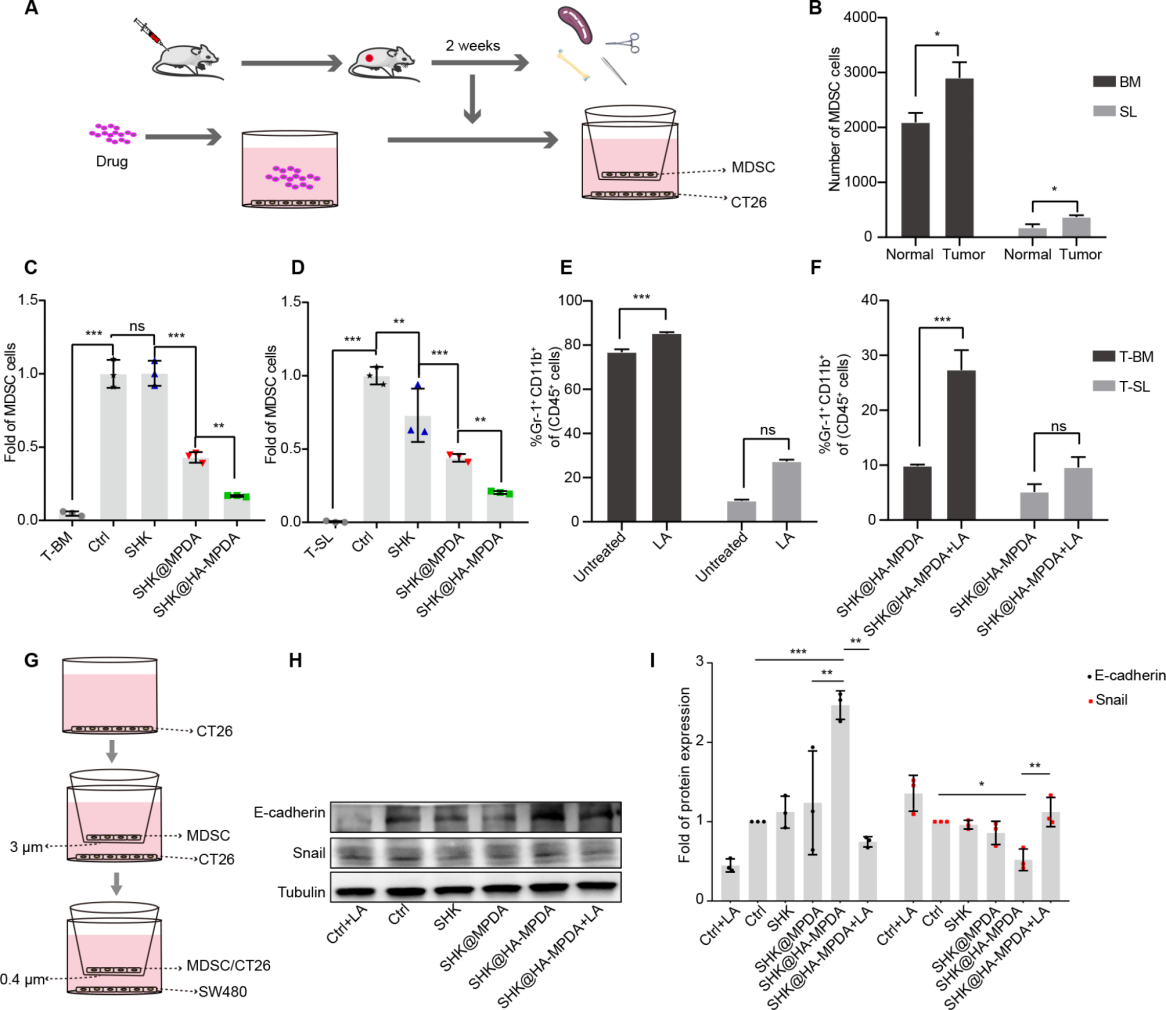
Downstream migration of MDSCs in the early colorectal cancer reversed the EMT in epithelial-like cells in vitro. (**A**) The schematic of MDSCs from the early colorectal cancer migrated to CT26 cells. (**B**) The MDSCs of bone marrow (BM) or spleen (SL) from normal or tumor mice were analyzed by flow cytometry. The migration capability of MDSCs from BM (**C**) or SL (**D**) of tumor mice after treatment with nanoparticles in CT26 cells. (**E**) LA intensified the migration capability of MDSCs from BM or SL of tumor mice in CT26 cells. (**F**) SHK@HA-MPDA inhibited the migration of MDSCs but was blocked by LA (LA 10 mM). (**G**) The schematic of MDSCs from tumor mice induced the epithelial-like cells EMT. (**H**) and (**I**) Western blot analysis and grayscale analysis of epithelial or mesenchymal markers in SW480 after co-incubating with a mix of MDSCs/CT26 cells. Data are presented as mean ± SD (n = 3). *P < 0.05, **P < 0.01, ***P < 0.001

Myeloid-derived suppressor cells (MDSCs) are pathologically activated and accumulate in the tumors; MDSCs are characterized by immunosuppressive and pro-tumorigenic activity [39], and play a key role in tumor metastasis [40–42]. To determine whether SHK can block MDSC migration to the tumors, the granulocytes from the bone marrow and spleen of the tumor-bearing mice were co-incubated with tumor cells (Fig. 7A and Fig. S3B). Our results suggested that the migration capacity of the MDSCs isolated from the tumor-bearing mice was stronger than the MDSCs from the normal mice (Fig. 7B). SHK@HA-MPDA treatment remarkably decreased the migration of the MDSCs isolated from the tumor-bearing mice (Fig. 7C and D). Interestingly, the lactate-treated CT26 cell also promoted the migration of MDSCs (Fig. 7E), but SHK@HA-MPDA treatment decreased the migration of MDSCs from the tumor-bearing mice (Fig. 7F). Furthermore, the co-cultures of MDSCs and CT26 cells were placed in an upper chamber and the SW480 cells in the lower chamber of a transwell cassette (Fig. 7G). As a result, the SW480 cells developed into a mesenchymal-like phenotype (Fig. 7H and I), suggesting the interplay between MDSCs and EMT; but the phenomenon can be reversed by SHK@HA-MPDA treatment.

### SHK-based nanotherapy in early liver metastasis of colorectal tumor model

**Fig. 8:**
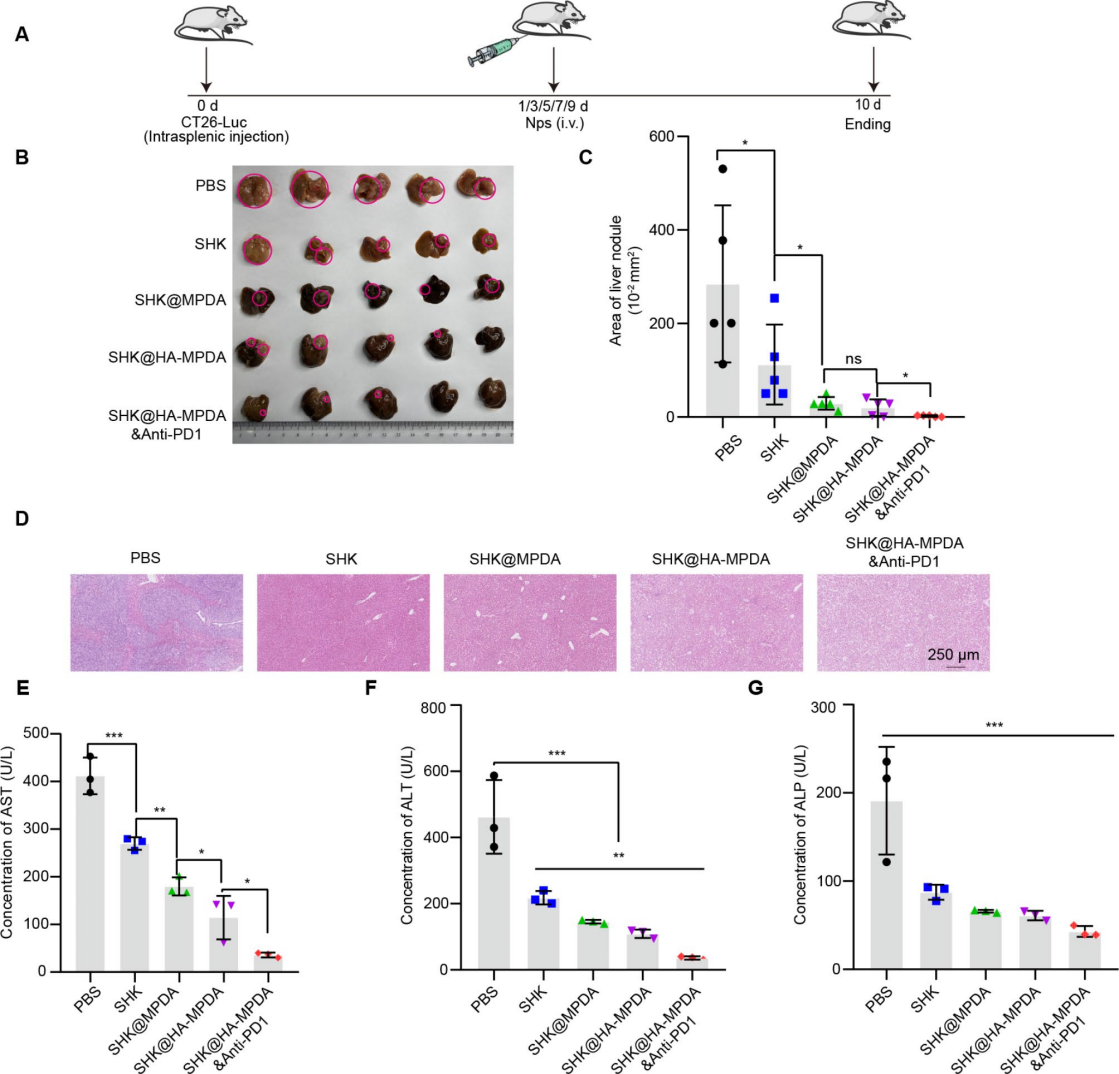
In vivo therapeutic efficacy of nanoparticles in a mouse model with early liver metastasis of colorectal cancer. (**A**) Therapeutic schedule. (**B**) The image of liver nodules of groups and the statistical analysis in (**C**). (**D**) The H&E strain of liver tissues. (**E–F**) Liver function index of serum (AST, ALT, and ALP). Data are presented as mean ± SD (n = 5). *P < 0.05, **P < 0.01, ***P < 0.001

Considering the severity of the advanced liver metastasis (late status), an early liver metastasis model was used to better demonstrate the treatment efficacy of SHK@HA-MPDA (Fig. 8A). As shown in Fig. 8B and C, SHK@HA-MPDA treatment significantly reduced the liver nodules compared to free SHK group. The levels of AST, ALT, and ALP and the histological examination results revealed that the SHK@HA-MPDA treatment improved the liver function of the mice (Fig. 8D). As shown in Fig. S6A and S6B the SHK@HA-MPDA treatment efficiently inhibited the expression of tetramer PKM2 and nuclear dimer PKM2 but upregulated E-cadherin and reversed EMT. It suggested the regulation of the crosstalk between glycolysis and EMT contributed to the remission of liver metastasis. The tumor immune microenvironment (TME) was remodeled and the activated anticancer immunity was characterized by the decreased immunosuppressive MDSC amount (Fig. S6C and Fig. S7), and the increased NK cells (Fig. S6D and S6E), CD8^+^ T cells (Fig. S6F), cytotoxic substances granzyme B and INFγ (Fig. S6G and S6H).

Importantly, SHK@HA-MPDA and anti-PD1 combination therapy yielded an obvious synergistic effect (Fig. 8E, F, and G) and showed the highest efficacy among all the groups.

### SHK-based nanotherapy in a mouse model with advanced liver metastasis of colorectal tumor

**Fig. 9:**
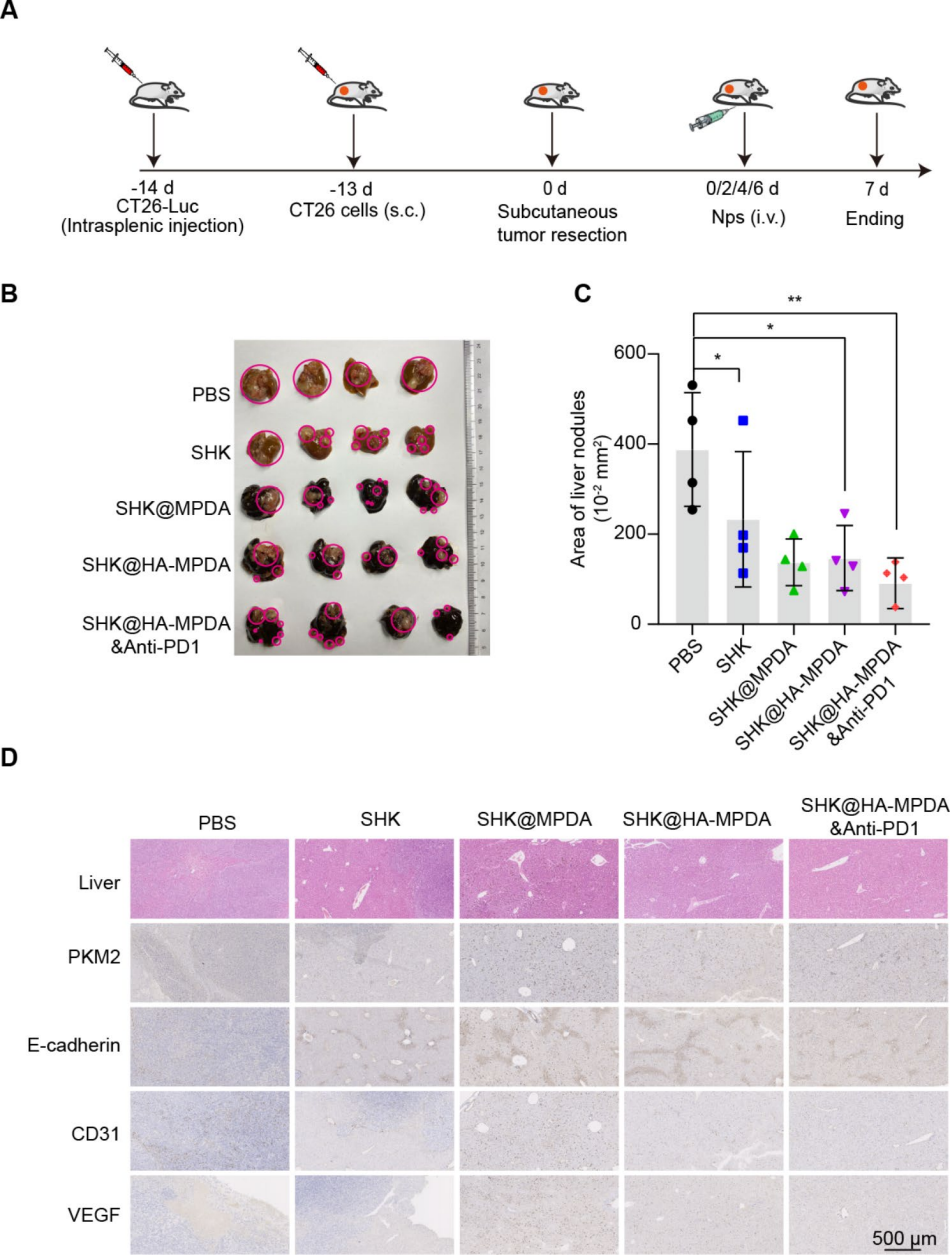
In vivo therapeutic efficacy of a mouse model with advanced liver metastasis of colorectal cancer and subcutaneous recurrence. (**A**) Therapeutic schedule. (**B**) The image of liver nodules of groups and the statistical analysis in (**C**). (**D**) The H&E strain and immunohistochemistry of PKM2, E-cadherin, CD31, and VEGF in liver tissues. Data are presented as mean ± SD (n = 4). *P < 0.05, **P < 0.01, ***P < 0.001

The CT26-Luc transplantation liver metastasis model was established (Fig. 9A). SHK@HA-MPDA treatment significantly reduced the metastasis nodules of the liver compared to the control group (Fig. 9B and C). Furthermore, as shown in Fig. 9D, the SHK@HA-MPDA group exhibited the anti-angiogenic effect, PKM2 inhibition, and EMT reversal; these mechanisms were responsible for liver metastasis remission.

**Fig. 10:**
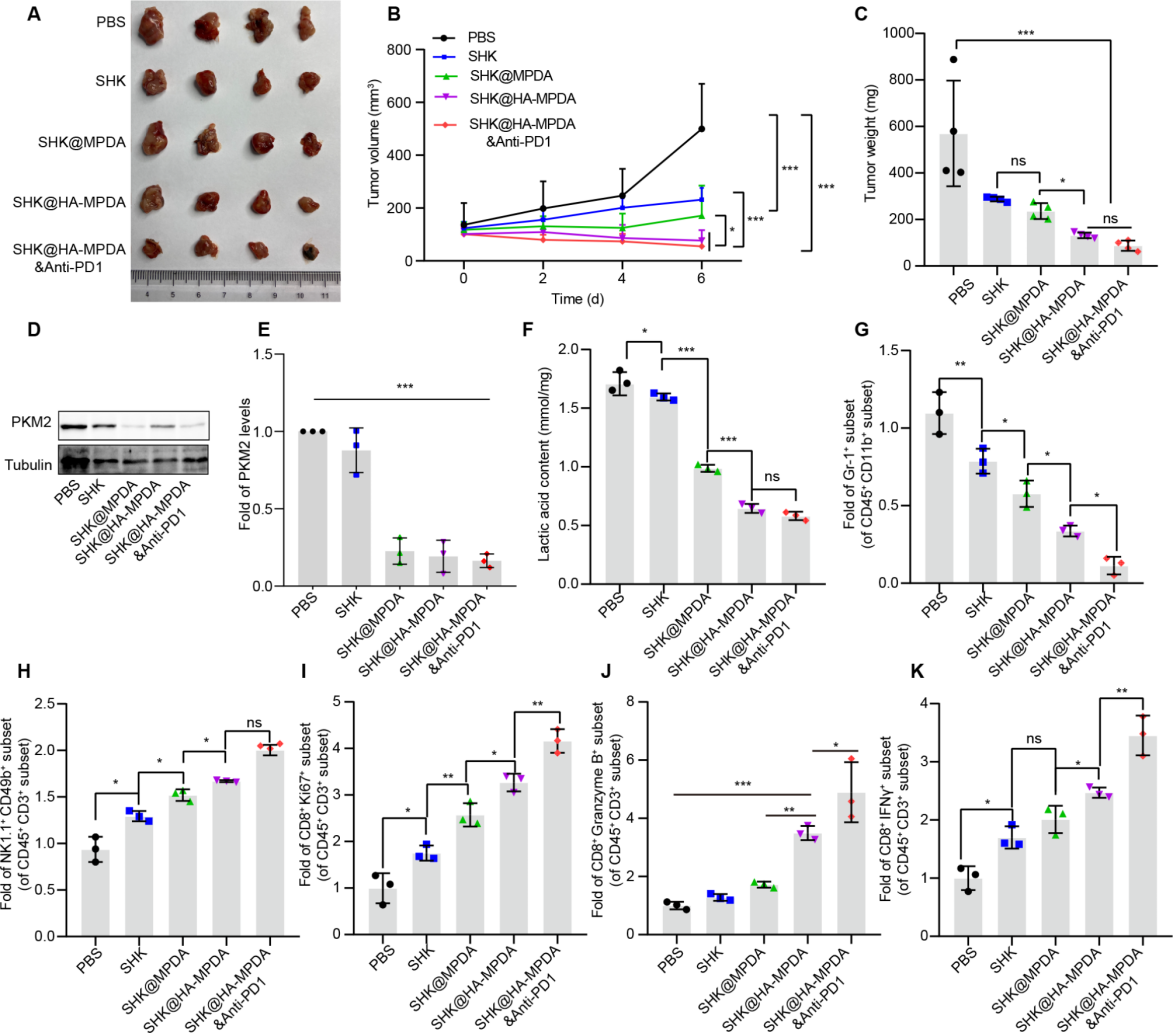
In vivo therapeutic efficacy of CT26 subcutaneous tumor in situ recurrence model. (**A**) The image of tumor volume of groups. (**B**) The tumor growth curve. (**C**) Statistical analysis of tumor weight after surgical of primary tumors. (**D**) and (**E**) The expression and grayscale analysis of PKM2 of tumor tissues. (**F**) The level of lactate in tumor tissues. The relative fold change of MDSCs (Gr-1^+^) (**G**), NK (NK1.1^+^CD49b^+^) (**H**), the CT8^+^T cells (CD8^+^Ki67^+^) (**I**), and cytotoxicity CD8^+^ T cells (CD8^+^Granzyme B^+^ and CD8^+^IFNγ^+^) (**J** and **K**). Data are presented as mean ± SD (n = 4). *P < 0.05, **P < 0.01, ***P < 0.001

The treatment efficacy was also evaluated in a postoperative recurrence model. The results showed that SHK@HA-MPDA treatment retarded the growth of the recurring tumor, with the highest efficacy among all the groups (Fig. 10A, B, and C). Similarly, the isolated tumor volume in a subcutaneous excision model at the experimental endpoint showed that SHK@HA-MPDA had improved antitumor efficacy (Fig. S8). SHK@HA-MPDA efficiently inhibited PKM2 expression and reduced the lactate level in the tumors (Fig. 10D, E, and F). As a consequence, the tumor immune microenvironment was remodeled (Fig. 10 and Fig. S9), as evidenced by the reduced immunosuppressive MDSC (Fig. 10G), the amplifying NK cells (Fig. 10H) and CD8^+^ T cells (Fig. 10I), and the increasingly released cytotoxic substances granzyme B and INF-γ (Fig. 10J and K).

Moreover, the anti-tumor effect of empty MPDA and anti-PD1 was evaluated in a postoperative recurrence model (Fig. S10A). The results showed the empty MPDA had a minor anti-tumor ability (Fig. S10B–J), which might be due to the regulation effect of empty MPDA on inflammation in the surgical site [43]. Tumor growth could be rested by suppressing the pro-inflammatory factors [44]. The treatment of anti-PD1 alone yielded an insufficient efficacy of tumor inhibition, compared to the combination therapy of anti-PD1 and SHK@HA-MPDA (Fig. S10B–J). It suggested that the CT26 colon tumor was insensitive to anti-PD1 therapy alone.

The preliminary safety evaluation showed no obvious lesions in the HE-stained sections of the main organs, and there was no significant change in body weight and organ coefficient (Fig. S11).

## Discussion

Colorectal cancer is the third largest killer of tumors which is characteristic of “cold” tumors attributed to immunodepression and develops multiple metastases, especially, liver and lymph nodes [1, 45]. Pharmacotherapy for metastatic colorectal cancer is unproductive, with poor clinical outcomes, because of the poor bioavailability, poor specificity, and multiple adverse effects [46, 47]. In this work, an immunometabolic strategy was developed by using a PKM2 inhibitor SHK, through which glycolysis and lactate production were suppressed and thus caused the reversal of anticancer immunity activation and EMT reversal, achieving a “one-stone-third-birds” effect.

We developed the HA-functionalized MPDA for the targeted delivery of SHK for CRLM. Cytoplasmic tetramer PKM2 is a critical factor that leads to the progression of CRC and CRLM [48, 49]. Patients with overexpression of tetramer PKM2 in CRC and CRLM are related to poor prognosis. Cytoplasmic tetramer PKM2 severs as a glycolytic enzyme for tumor metabolism, but after translocation into the nucleic, the dimer PKM2 induces nuclear ectopic elaborated as a protein kinase [50, 51], binding to histone H3 and phosphorylates histone H3 at T11 which is responsible for the dissociation of HDAC3 from MYC promoter regions [25]. The SHK@HA-MPDA nanosystem efficiently inhibited tetramer PKM2 and further reduced lactate and ATP production, and limited tetramer PKM2-driven nuclear ectopic as well.

Aggressive glycolysis fuels the tumor progression and lactate flux that reshapes the tumor microenvironment [52]. Our previous work demonstrated the interplay of lactate and tumor-associated macrophage (TAM) polarization and their contribution to tumor immunosuppression [53]. Besides, CT26 colorectal tumor is known for its “immune-cold” nature, and its treatment can be improved by remodeling the tumor immune microenvironment [54]. Therefore, SHK@HA-MPDA not only directly killed cancer cells and induced ICD but also inhibited lactate-induced immunosuppression.

Notably, extracellular lactate also serves as a protumor signal molecule. For example, TGFβ can be activated by lactate through a pH-dependent manner, which induces LDH5 expression via HIF1α and contributes the myofibroblast differentiation [38]. Besides, the TGFβ/EGF pathway mediates the nuclear ectopic of PKM2, thereby leading to EMT that promotes tumor metastasis [55]. The glycolysis-related enzyme is overexpressed in mesenchymal-like cells [56, 57], indicating the crosstalk between metabolic change and EMT in the tumor. Our results revealed that the tumor cells treated by TGFβ, thus being mesenchymal-like, produced more lactate than the untreated cells, which suggested that mesenchymal-like cells were avid for glycolysis compared to epithelial-like cells. In turn, lactate secreted from the mesenchymal-like cells further induces EMT in the epithelial-like cells. Therefore, the SHK@HA-MPDA nanosystem can inhibit glycolytic lactate production and subsequently reverse EMT.

MDSCs are the immunosuppressive immune cells in the tumor and promote angiogenesis, thus creating a premetastatic environment [58, 59]. The intratumor MDSCs actively promote cancer cell invasion by inducing EMT in the early state [60]. The SHK@HA-MPDA nanosystem can also act on the MDSCs by reducing their recruitment in the tumor.

## Conclusions

In this work, we designed a targeting SHK@HA-MPDA nanosystem for CRLM treatment by targeting lactate-based immnuometabolism and thus remodeling the tumor immune microenvironment. Furthermore, the SHK@HA-MPDA nanosystem reversed EMT of the cancer cells by suppressing lactate production and thus showed a substantial treatment efficacy in CRLM. The shikonin nanomedicine provides a promising “one-stone-three-birds” therapeutic strategy for CRLM.

## Materials and methods

### Materials

Shikonin (SHK) was purchased from DASF Biotechnology Co., Ltd (Nanjing, China). Dopamine hydrochloride, 1,3,5-trimethylbenzene (TMB), and Pluronic F-127 were purchased from Sigma-Aldrich (St. Louis, USA). Hyaluronic acid (HA, 10 kD) kit and Mycoplasma test kit were purchased from Meilun Biotechnology (Dalian, China). Dulbecco’s modified Eagle’s Medium (DMEM) cell culture medium was from Gibco (Thermo Fisher Scientific, Waltham, USA) and fetal bovine serum (FBS) was from GeminiBio (West Sacramento, USA). Collagenase IV and hyaluronidase were purchased from Yeasen Biotechnology Co., Ltd (Shanghai, China). Antibodies of PKM2, C-MYC, E-cadherin, Snail, Vimentin, α-SMA, HMGB1, TGF-β, and Histone H3 were purchased from Cell Signaling Technology (Boston, USA). Antibodies of E-cadherin EGF, HIF1α, CD44, and PDL1 were purchased from Abcam (Cambridge, UK). Vinculin, MCT4, GAPDH, and Tubulin were purchased from Absin (shanghai, China). A lactic acid assay kit was purchased from Jiancheng Bioengineering Co., Ltd. (Nanjing, China). An enhanced ATP assay kit was from the Beyotime Institute of Biotechnology (Haimen, China). The mouse lymphocyte separation medium was from Shanghai Dakewe Biotech Co., Ltd (Shanghai, China). Purified rat anti-mouse CD16/CD32 (mouse BD Fc block), FITC or APC CY7 rat anti-mouse CD45, APC or Percp-cy5.5 rat anti-mouse CD11b, Percp-cy5.5 or PE rat anti-mouse Gr-1, BV421 rat anti-mouse Ki67, APC rat anti-mouse CD8, APC-CY7 rat anti-mouse IFNγ, PE rat anti-mouse Granzyme B, FITC rat anti-mouse CD49b and APC rat anti-mouse NK1.1 were from Biolegend (San Diego, USA). Anti-mouse PD-1 was from BioXcell (Lebanon, USA). Other analytical grade reagents were from Sinopharm Chemical Reagent Co., Ltd. (Shanghai, China).

### Cell lines

The murine colorectal cancer cells (CT26), the CT26 cells with stable expression of firefly luciferase reporter (CT26-Luc), human normal colon epithelial cells (NCM460), murine colorectal cancer cell (C26), human colorectal cancer cells (HCT116, SW480, HCT8), paclitaxel-resistant HCT8 cells (H/T), and human umbilical vein endothelial cell (HUVEC) were obtained from the Shanghai Cell Bank of Chinese Academy of Sciences (Shanghai, China). CT26, CT26-Luc, and C26 were cultured in RPMI-1640 medium with 10% FBS and 1% penicillin-streptomycin (Beyotime, Shanghai, China). HCT116, HCT8, H/T, and HUVEC were cultured in Dulbecco’s modified Eagle media (DMEM) with 10% FBS and 1% penicillin-streptomycin. All cells were maintained in a humidified 5% CO_2_ incubator at 37 °C.

### Animals

The Balb/c mice (6–8 weeks) were provided by Shanghai Laboratory Animal Center (Shanghai, China) and housed at the specific pathogen-free (SPF) care facility. All the animal experimental procedures were approved by the Institutional Animal Care and Use Committee (IACUC), Shanghai Institute of Materia Medica, Chinese Academy of Sciences.

### Colorectal cancer liver metastasis model

The liver metastasis model in the Balb/c mice was developed by intrasplenic injection of the tumor cells as described in the previous report [61]. Briefly, the mice were anesthetized and placed in the right lateral decubitus position. The left flank through the skin and peritoneum was externalized with a 1–2 cm incision, exposing the spleen. In total, approximately 1 × 10^6^ CT26-Luc cells were resuspended in RPMI 1640 without FBS and injected into the spleen with a 33-gauge needle. The abdominal wall and skin were then sutured. The mice were humanely euthanized at a predetermined endpoint.

### Tumor recurrence model with subcutaneous transplantation

Approximately 1 × 10^6^ CT26 cells in 100 µL of RPMI 1640 were injected subcutaneously into the right flank. The tumors were removed when grown into 200–300 mm^3^. All mice were humanely euthanized according to the following criteria significant body-weight loss (> 20%), tumor size exceeding 2,000 mm^3^, or when tumors ulcerated.

### Database

The GSE21510 database from Gene Expression Omnibus (GEO) was used to verify the differential expression of PKM2 in normal tissues, CRC primary tumor, and liver metastasis. The correlation between the PKM2 expression and its clinical manifestations was validated in the Cancer Genome Altas (TCGA) database. All expression profiling data in this study were obtained from GEO (https://www.ncbi.nlm.nih.gov/geo/) and TCGA (https://portal.gdc.cancer.gov/).

### Expression difference of PKM2 between normal liver and colorectal liver metastasis

The normal cells (HUVEC and NCM460) and colorectal cells (CT26, C26, CaCO2, HCT116, HCT8, and H/T) were used to analyze the difference in PKM2 expression by western blotting. Furthermore, the PKM2 expression in the colorectal cancer liver metastasis model or the normal mouse liver was examined through immunohistochemistry.

### Preparation of the MPDA

The MPDA was prepared using a modified method from a previous report [26]. In short, 100 mg Pluronic F127 and 50 mg hydrochloride dopamine were dissolved in 10 mL of water and ethanol (1:1) mixture. Then, 200 µL TMB was dripped slowly into the solution at a stirring rate of 500 rpm at room temperature. After reaction for 30 min, the mixed solution continued to react for 12 h with 5.0 mL of concentrated ammonia (NH_4_OH). Finally, the MPDA nanoparticles were centrifuged at 12,000 rpm and washed with 50% ethanol 3 times, and then resuspended in ultra-pure water.

### Preparation of SHK@MPDA

Due to plentiful aromatic rings in MPDA, it is easy to load SHK *via* π–π stacking and/or hydrophobic–hydrophobic interactions. Pluronic F127 (100 mg) was dissolved in 10 mL of 30% ethanol aqueous solution, and then 18.5 mg MPDA was added to the solution under stirring. SHK dissolved in 1 mL of a mixture solution of chloroform and ethanol (1:2) was dropped into the above reaction mixture for reaction for 12 h. The SHK@MPDA nanoparticles were separated by centrifugation at 12,000 rpm and resuspended in ultra-pure water.

### Preparation of SHK@HA-MPDA

The MPDA was modified with hyaluronic acid (HA). In brief, HA (200 mg) was dissolved in 10 mL of formamide at 50 °C and, after cooled to room temperature, it was added to 15 ml formamide containing 520 mg EDC and 310 mg NHS for reaction for 30 min. The activated HA was dropped slowly into 1 mL of ethylenediamine on the ice, and then stirred for 3 h at room temperature. Subsequently, sufficient pre-cooled acetone was added to the reaction system and after overnight the precipitation was collected by using centrifugation and washed with acetone. Finally, the precipitation was dialyzed in water for 48 h and freeze-dried. The amino-functionalized HA was used to modify MPDA via Schiff base reaction at a reaction ratio of 4:1 at pH 8.5 for 2 h. The thus-formed SHK@HA-MPDA nanoparticles were purified by centrifugation at 12,000 rpm and resuspended in ultra-pure water.

### Characterization of SHK@HA-MPDA

The size, polydispersity (PDI), and zeta potential of SHK@HA-MPDA nanoparticles were measured by a Zeta Sizer Nanoparticle Analyzer (Malvern Panalytical, Malvern, UK). The morphological observation of SHK@HA-MPDA was carried out by transmission electron microscopy (TEM). The drug encapsulation efficiency (EE%) and drug-loading capacity (DL%) of SHK@HA-MPDA were determined by high-performance liquid chromatography (HPLC) (1260 Infinity, Agilent Technologies, Santa Clara, USA) equipped with Agilent-C18 (5 μm, 250 mm × 4.6 mm). The chromatographic conditions for SHK determination were listed as follows: mobile phase composed of methanol (90%) and phosphoric acid (0.01%) aqueous solution, flow rate of 1 mL/min, and detection wavelength of 516 nm. The in vitro drug release was evaluated by a dialysis method using a dialysis membrane (MWCO 8,000–14,000 Da) in PBS (pH 5.0) containing 0.5% (w/v) SDS with 150 rpm/min shaking at 37 °C. The released drugs were quantified by HPLC as described above. The serum stability of SHK@HA-MPDA was evaluated by suspending them in PBS containing 10% new-born bovine serum (Zhejiang Tianhang Biotechnology Co., Ltd, Hangzhou, China) with a general shaking at 150 rpm at 37 °C. The size of particles at a specified time was measured by a Zeta Sizer Nanoparticle Analyzer.

### Photothermal properties of the MPDA NPs

The properties of photothermal conversion of MPDA NPs were investigated by monitoring the temperature elevation under irradiation of an 808 nm NIR laser at 2.0 W/cm^2^. Various concentrations of 1.0 mL MPDA nanoparticles (1000, 2000, 4000, and 8000 µg/mL) were exposed to an 808 nm laser for 10 min. The temperature of the solutions was recorded by using an infrared thermal imaging instrument (New Industries Optoelectronics Tech Co., Ltd, Changchun, China) every 10 s. Subsequently, the different power of the laser (0.5, 0.75, 1.0, 1.5, 2.0 W/cm^2^) was used to evaluate the photothermal performance of MPDA at a fixed concentration of 8000 µg/mL, and thermal imaging was recorded. Furthermore, after the laser was turned off, the temperature variation of the solution was recorded every 10 s.

### Cellular uptake efficiency test

The CT26 cells were seeded in a 12-well plate with 1 × 10^5^ cells/well and cultured for 12 h. The cells were treated with equivalent 5-FITC dye-encapsulated MPDA NPs for 4 h and the intracellular fluorescence was detected by flow cytometry (ACEA NovoCyte 3000, Agilent, USA). Furthermore, the optimal ratio of MPDA and HN_2_-HA was evaluated by uptake efficiency. Besides, the cells were treated with equivalent coumarin-6 dye-encapsulated MPDA NPs and fluorescent images were obtained by the inverted fluorescence microscope (CARL ZEISS, Oberkochen, Germany).

### Mycoplasma test

The mycoplasma of CT26 cells and NCM460 cells was tested by Hoechst fluorescence staining, which is based on the DNA-dye intercalation—when the cells are stained with Hoechst 33,258, and the contaminated cultures can be detected by the bright, punctate cytoplasmic staining of the mycoplasma DNA that contains abundant A-T with high affinity to Hoechst 33,258 [62]. The sterile climbing tablets were put into a 24-well plate. CT26 cells or NCM460 cells were seeded on climbing tablets and cultured for 48 h. The adherent cells were washed with PBS and fixed by fixed liquid for 20 min. Next, the cells were stained by Hoechst 33,258 (Meilun Biotechnology Co., Ltd., Dalian, China). The fluorescent images were obtained by confocal microscopy (Olympus FV100, Japan).

### In vitro cytotoxicity test

The CT26 cells were seeded in a 96-well plate with 5 × 10^3^ cells/well and cultured for 12 h. MTT assay was used to evaluate the cytotoxicity of cells treated with free SHK, SHK@MPDA, SHK@HA-MPDA, and MPDA for 48 h, followed by a standard MTT assay.

The NCM460 cells were seeded in a 96-well plate with 5 × 10^3^ cells/well and cultured for 12 h. MTT assay was used to evaluate the cytotoxicity of cells treated with SHK@HA-MPDA for 48 h, followed by a standard MTT assay.

### Lactate and ATP assay

A lactic acid assay kit was used to detect the concentration of lactate in the CT26 cell culture medium or the tumor tissues (Jiancheng Bioengineering Co., Ltd., Nanjing, China). The cells were cracked for ATP quantification according to the manufacturer instruction (Beyotime, Shanghai, China).

### ICD and activate immune-related cells

The cells were seeded in a 12-well plate for 12 h and then treated with SHK (1.5 µM), SHK@MPDA (1.5 µM), SHK@HA-MPDA (1.5 µM), SHK@HA-MPDA&LA (1.5 µM) for 8 h. The cells were collected for both western blotting and flow cytometry analysis.

The Bone marrow-derived mononuclear cells were collected from the femur and tibia of the Balb/c mice using a standard protocol. Subsequently, bone marrow-derived dendritic cells (BMDCs) were induced by being treated with GM-CSF (20 ng/mL) and IL4 (10 ng/mL) for 4 days. Then, the BMDCs were co-cultured with the CT26 cells pretreated free SHK or SHK@HA-MPDA (1.5 µM) for 10 h and stimulated with LPS (200 ng/mL) or LA (10 mM) simultaneously. The mature DCs (CD80^+^CD86^+^ subset) were analyzed by flow cytometry.

The CT26 cells were pretreated with free SHK or SHK@HA-MPDA (1.5 µM) for 24 h, and then the cells were co-cultured with BMDC for 24 h accompanied with LA (10 mM) or LPS (200 ng/mL). The sensitized BMDCs were subsequently co-cultured with the splenic lymphocytes isolated from the healthy mice using a lymphocyte separation medium (Shanghai Dakewe Biotech Co., Ltd, Shanghai, China) for 3 days. The proliferative T cells (CD8^+^Ki67^+^) and cytotoxic T lymphocytes (CD8^+^IFNγ^+^, CD8^+^Granzyme B^+^) were analyzed by flow cytometry.

### Cell colony-forming assay

The CT26 cells were seeded in a 12-well plate with 1 × 10^5^ cells/well for 12 h and treated with SHK (1.0 µM), SHK@MPDA (1.0 µM), SHK@HA-MPDA (1.0 µM) for 24 h. The live cells were collected and seeded in a 6-well plate with 3,000 cells/well for 12 days. The fresh culture medium was replaced in the 7th days and 10th days. At the endpoint, the cells were washed with sterile PBS 3 times and fixed with 4% paraformaldehyde for 15 min, following dyed with 0.5% purple crystal, and washed with sterile PBS.

### Scratch assay

The CT26 cells were seeded in a 6-well plate with 1 × 10^6^ cells/well for 12 h and scratched with a 200 µL pipette tip. The cells were then washed with sterile PBS and treated with SHK (1.0 µM), SHK@MPDA (1.0 µM), or SHK@HA-MPDA (1.0 µM) for 24 h in the serum-free 1640 medium. The scratch distance was obtained using an inverted fluorescence microscope.

### Migration assay and matrigel invasion assay

The CT26 cells were seeded in a 6-well plate with 1 × 10^6^ cells/well and treated with SHK (1.0 µM), SHK@MPDA (1.0 µM), or SHK@HA-MPDA (1.0 µM) for 24 h. Afterward, the cells were collected and re-seeded at a density of 1 × 10^5^ cells/well in the upper chamber of a transwell plate (pore size of 8.0 μm) for 48 h. The migrating cells in the bottom chamber were dyed with 0.5% purple crystal and washed with PBS. The image figures were obtained by an inverted fluorescence microscope.

For Matrigel assay, the upper chamber in a transwell plate was coated with matrigel (100 µL) and then CT26 cells pre-treated with SHK (1.0 µM), SHK@MPDA (1.0 µM), or SHK@HA-MPDA (1.0 µM) were seeded with 1 × 10^5^ cells/well for 48 h. The invasion cells in the bottom chamber were dyed with 0.5% purple crystal and washed with PBS. The image figures were obtained by an inverted fluorescence microscope.

### Inhibition of MDSC migration further reversed EMT

It is reported that the MDSC has a potent capability of migration in the tumor-bearing mice in an early stage [39]. Thus, the Balb/c mice were inoculated subcutaneously with 1 × 10^6^ CT26 cells for 2 weeks. The single-cell suspensions from the bone marrow or spleen of the tumor-bearing mice were collected using a standard protocol. Then, the single cell suspension was placed in the upper chamber of the transwell (pore size of 3.0 μm) and co-cultured with CT26 cells in the bottom chamber pre-treated SHK (1.5 µM), SHK@MPDA (1.5 µM), or SHK@HA-MPDA (1.5 µM) for 24 h. Whereafter, the migrated cells in the bottom chamber from the upper chamber and CT26 cells were collected, and next the number of MDSCs (CD11b^+^Gr-1^+^) was analyzed by flow cytometry.

To evaluate the effect of lactate on MDSC, CT26 cells were pretreated with SHK@HA-MPDA for 24 h and placed in the bottom chamber. CT26 cells were then co-cultured with single-cell suspensions from the bone marrow or spleen of tumor-bearing mice in the upper chamber with lactate (10 mM). Afterward, the number of MDSCs (CD11b^+^Gr-1^+^) in the bottom chamber was analyzed by flow cytometry.

To investigate the crosstalk of MDSC and EMT, CT26 cells were pretreated with SHK (1.5 µM), SHK@MPDA (1.5 µM), and SHK@HA-MPDA (1.5 µM) for 24 h in the bottom chamber of transwell (pore size of 3.0 μm), subsequently co-cultured with single-cell suspensions from the bone marrow of tumor-bearing mice for 24 h in the upper chamber. The cocktail of CT26 cells and MDSC from the above bottom chamber were collected to the upper chamber of a new transwell (pore size of 0.4 μm), and then continued to co-culture with SW480 cells in the bottom chamber of the new transwell for 48 h. Finally, the protein of SW480 cells was collected and analyzed by western blot.

### In vivo photothermal image

The Balb/c mice were subcutaneously injected with 2 × 10^6^ CT26-Luc cells for 7 days and the mice were randomly divided into PBS, SHK@MPDA (5 mg/kg), and SHK@HA-MPDA (5 mg/kg) (n = 3). The tumor was irradiated by a laser of 808 nm and 2 W/cm^2^, and then, the local temperature was recorded every 5 s during 100 s at 8, 12, 24, 30, and 48 h after tail intravenous administration. Besides, the photothermal images of the 100 s were obtained by the infrared imager.

### Establishment of early liver metastasis model of colorectal cancer

The Balb/c mice were intrasplenically inoculated with 1 × 10^6^ CT26-Luc cells to establish liver metastasis of colorectal cancer described above and randomly divided into SHK (5 mg/kg), SHK@MPDA (5 mg/kg), SHK@HA-MPDA (5 mg/kg), and SHK@HA-MPDA&anti-PD1 (5 mg/kg + 10 mg/kg) (n = 5). The mice were given tail vein injection of nanosystem and intraperitoneal injection of anti-PD1 on days 1, 3, 5, 7, and 9. At the endpoint, the mice were humanely sacrificed and the organs were collected for further study.

### Establishment of advanced liver metastasis model of colorectal cancer accompanied by subcutaneous excision

The Balb/c mice were intrasplenically inoculated with 1 × 10^6^ CT26-Luc cells to establish liver metastasis of colorectal cancer described above. Then every mouse was subcutaneously injected with 1 × 10^6^ CT26 cells one day later. After 13 d, the subcutaneous tumors were removed by surgery. The groups of SHK (5 mg/kg), SHK@MPDA (5 mg/kg), SHK@HA-MPDA (5 mg/kg), and SHK@HA-MPDA&anti-PD1 (5 mg/kg + 10 mg/kg) (n = 4) were injected *via* the tail vein of nanosystem and intraperitoneally injected with the anti-PD1 on day 0. At last, 0.5 mL of blood was obtained through orbital venous plexus blood collection after anesthesia. Subsequently, the main organs and tumors were collected for pathological analysis.

### Establishment of subcutaneous excision model of colorectal cancer

The Balb/c mice were subcutaneously injected with 2 × 10^6^ CT26 cells. After 8 d, the subcutaneous tumors were removed by surgery. The groups of MPDA (50 mg/kg), Anti-PD1 (10 mg/kg), SHK@HA-MPDA (5 mg/kg), and SHK@HA-MPDA&anti-PD1 (5 mg/kg + 10 mg/kg) (n = 5) were injected *via* the tail vein of nanosystem and intraperitoneally injected with the anti-PD1 on day 1, 3, 5, 7, 9, and 11. At the endpoint, the mice were humanely sacrificed and the subcutaneous tumors were collected.

### Statistical analyses

All data were analyzed through GraphPad Prism 8.0 software. The results were represented as the mean ± SD (n ≥ 3) and statistical analysis was performed by Student’s t-test or one-way ANOVA (*P < 0.05, **P < 0.01, and ***P < 0.001.

## Electronic supplementary material

Below is the link to the electronic supplementary material.

Supplementary Material 1

**Supplementary Material 2: Fig. S1.** The confocal images of CT26 cells and NCM460 cells. **Fig. S2.** Preliminary evaluation regulating EMT of SHK in a CRLM model. (A) and (B) The expression and grayscale analysis of E-cadherin of epithelial or mesenchymal markers in CT26 and NCM460 cells. (C) Expression of epithelial or mesenchymal markers in CT26 cells after treatment with free SHK. (D) The grayscale analysis of (C). (E) Therapeutic schedule. (F) The expression of E-cadherin of liver tissues in normal and tumor mice. (G) and (H) Expression and grayscale analysis of epithelial or mesenchymal markers of normal or tumor mice. (I) and (J) Western blot analyzed free SHK-regulated EMT in a CRLM model and grayscale analysis of EMT-related makers. (K) Grayscale analysis of Fig. 6G. (L) Grayscale analysis of Fig. 6H. **Fig. S3.** Flow cytometry gate diagram of CTLs (A) and MDSCs (B) in vitro. **Fig. S4.** LA inducing EMT depending on TGF/EGF in an EMT model of SW480 cells. (A) and (B) Expression and grayscale analysis of E-cadherin in colorectal cancer cells. (C) and (D) Western blot analysis of epithelial or mesenchymal markers in mesenchymal-like cells with or without TGFβ (10 ng/mL) and SB431542 (5 µM). (E) The LA production of mesenchymal-like cells and epithelial-like cells (TGFβ: 10 ng/mL). (F) and Western blot analysis of LA depended on pH-stimulated TGF/EGF inducing EMT in SW480 cells (LA: 20 mM; SB431542: 5 µM). (G) Grayscale analysis of (F). **Fig. S5.** The profiles of SHK@MPDA@HA nanosystem. (A–D) The photothermal performance of MPDA in vitro. The temperature changes curve with 808 NIR irradiation of CT26 subcutaneous tumor after treatment with SHK@MPDA (E) and SHK@MPDA@HA (F). The temperature changes curve (G) and image (H) of CT26 subcutaneous tumor with 808 NIR irradiation at 30 h. **Fig. S6**. Reversion of EMT and remodeling tumor immunodepression microenvironment in early liver metastasis of colorectal cancer. (A) Western blot analysis of epithelial or mesenchymal markers of liver tissues. (B) Grayscale analysis of (A). (C) The relative fold change of the MDSCs subset (CD11b^+^Gr-1^+^). (D) and (E)The relative fold change of the NK subset (CD49b^+^NK1.1^+^, CD49b^+^NK1.1^+^IFNγ^+^). (F) The relative fold change of the CT8^+^T cells (CD8^+^Ki67^+^) and (G and H) cytotoxicity CD8^+^ T cells (CD8^+^Granzyme B^+^ and CD8^+^IFNγ^+^). **Fig. S7.** Flow cytometry gate diagram of MDSCs (A), NK cells (B), and CTLs (C) in a mouse model with early liver metastasis of colorectal cancer. **Fig. S8** The isolated tumor volume in a subcutaneous excision model at end point of experiment. **Fig. S9.** Flow cytometry gate diagram of MDSCs (A), NK cells (B), and CTLs (C) of CT26 subcutaneous tumor in situ recurrence model. **Fig. S10** In vivo therapeutic efficacy of CT26 subcutaneous excision model. (A) Therapeutic schedule. (B) The tumor growth curve. (C) Statistical analysis of tumor weight after surgical of primary tumors. (D) The image of tumor volume of groups. (E) Statistical analysis of the isolated tumor volume of subcutaneous excision model at end point of experiment. (F–J) Individual tumor growth curves of PBS, MPDA, Anti-PD1, and SHK@HA-MPDA, and SHK@HA-MPDA&Anti-PD1 groups. **Fig. S11.** (A–C) Preliminary safety evaluation

## Data Availability

All data generated or analyzed during this study are included in this published article and its supplementary information file.
