# Supplementary material for: Regulating lactate-related immunometabolism and EMT reversal for colorectal cancer liver metastases using shikonin targeted delivery
Source: J Exp Clin Cancer Res. 2023 May 10;42:117. doi: 10.1186/s13046-023-02688-z (PMC10170793; doi:10.1186/s13046-023-02688-z)

Original western blot bands

Regulating lactate-related immunometabolism and EMT reversal for colorectal cancer liver metastases using shikonin targeted delivery

Li Long[1, 2, 3], Wei Xiong[1, 2, 3], Fenwang Lin[4], Jiazhen Hou[2, 3, 5], Guihua Chen[1, 2, 3], Taoxing Peng[2, 3], Rui Wang [2, 3], Qin Xu[1, *], Yongzhuo Huang[1, 2, 3, *]

Appendix 1. Un-chopped images of Fig. 1D.

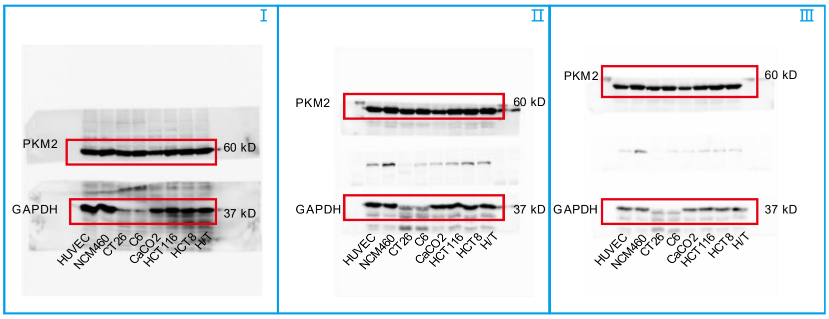

Appendix 2. The un-chopped images of Fig. 3A.

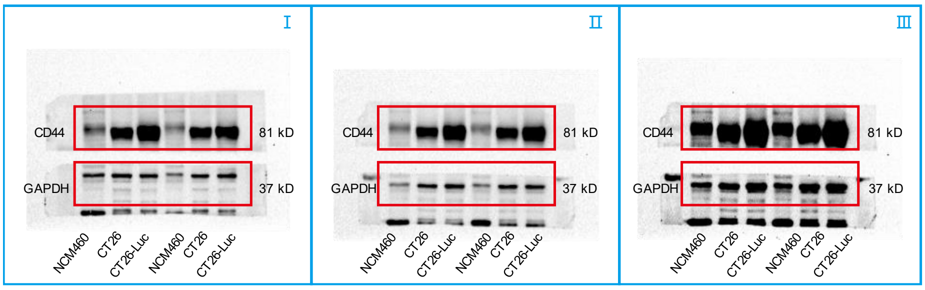

Appendix 3. The un-chopped images of Fig. 4F.

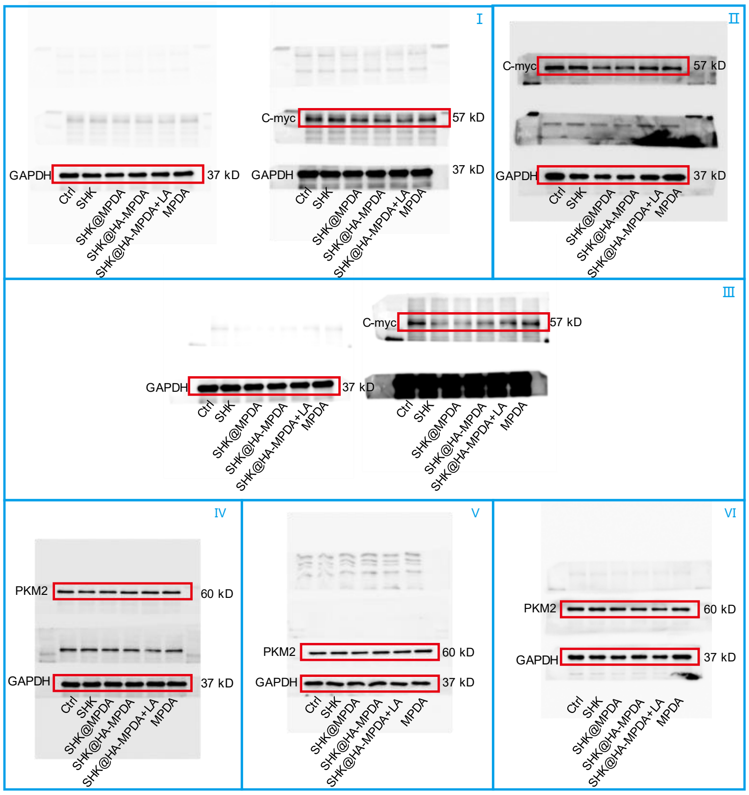

Appendix 4. The un-chopped images of Fig. 5B.

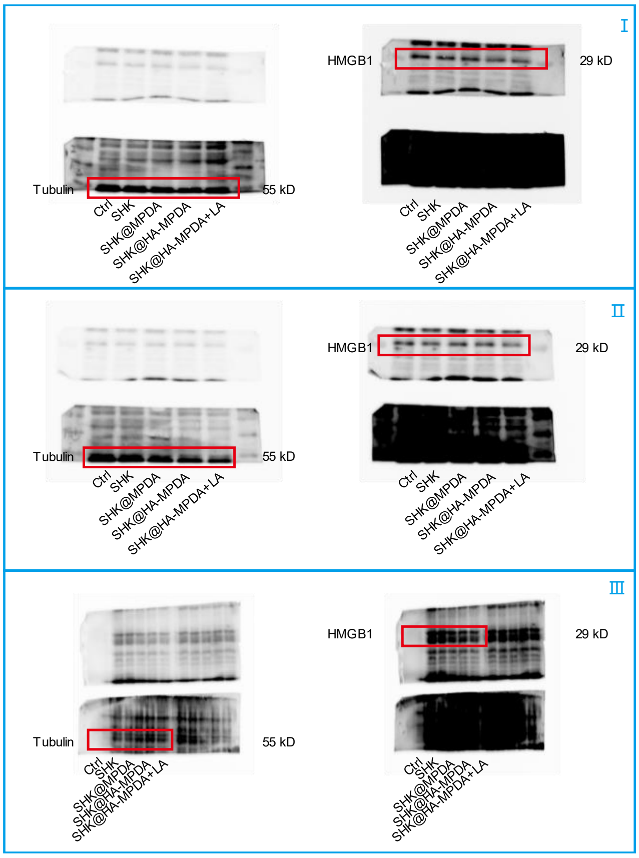

Appendix 5. The un-chopped images of Fig. 6G.

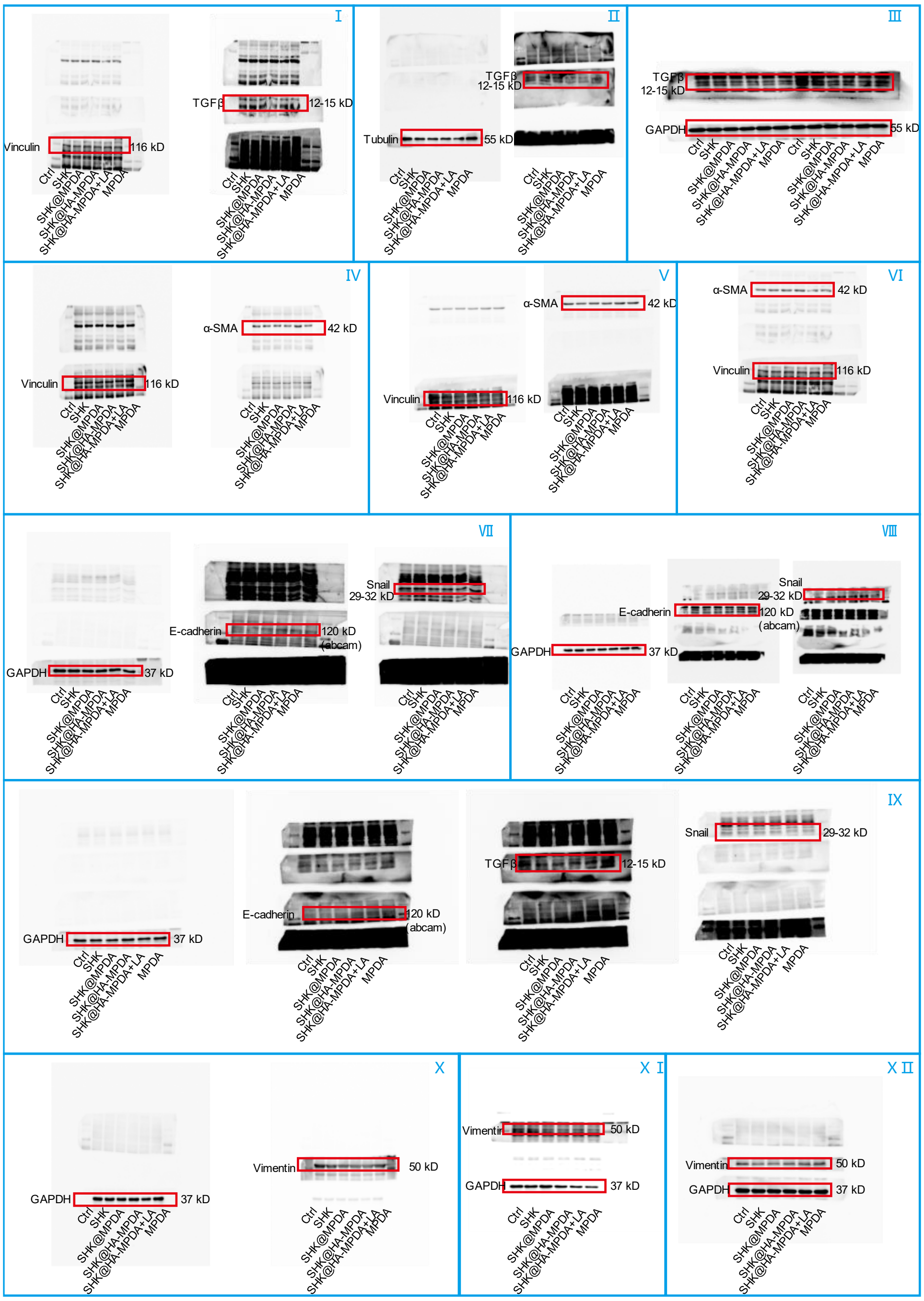

Appendix 6. The un-chopped images of Fig. 6H.

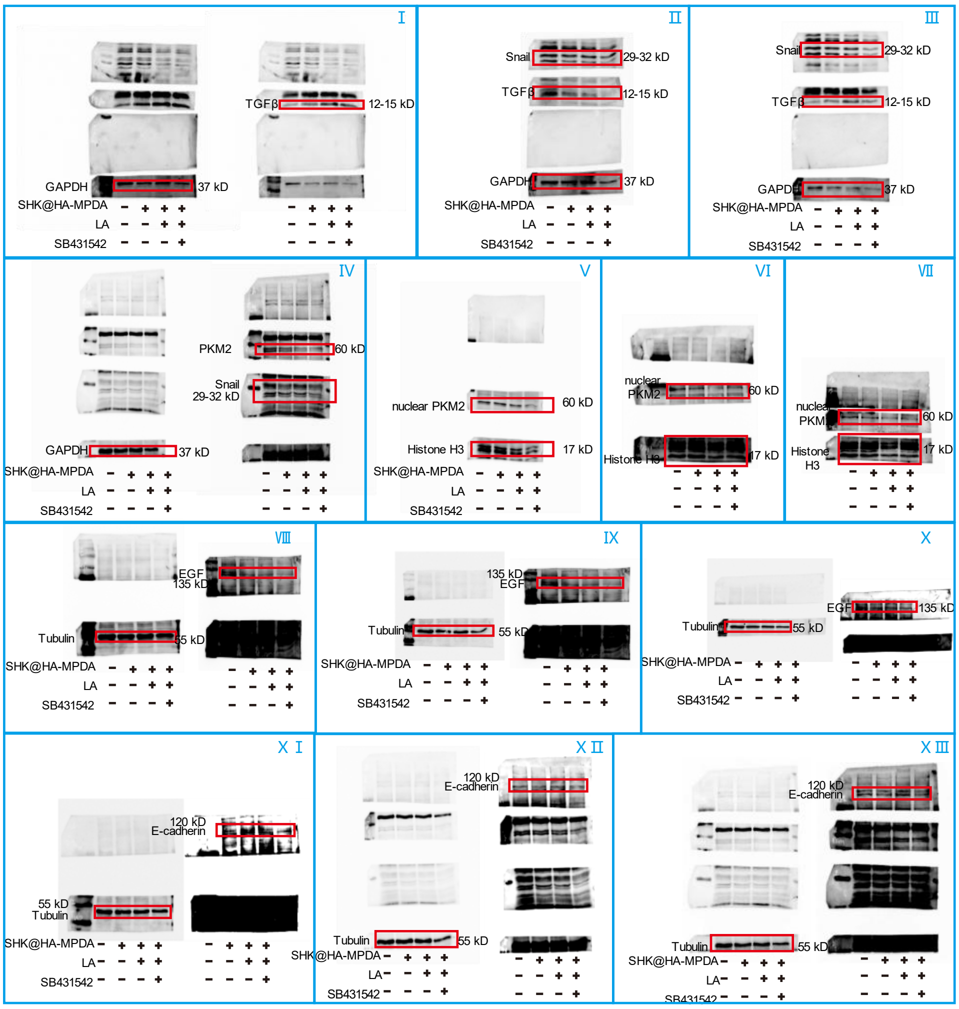

Appendix 7. The un-chopped images of Fig. 7H.

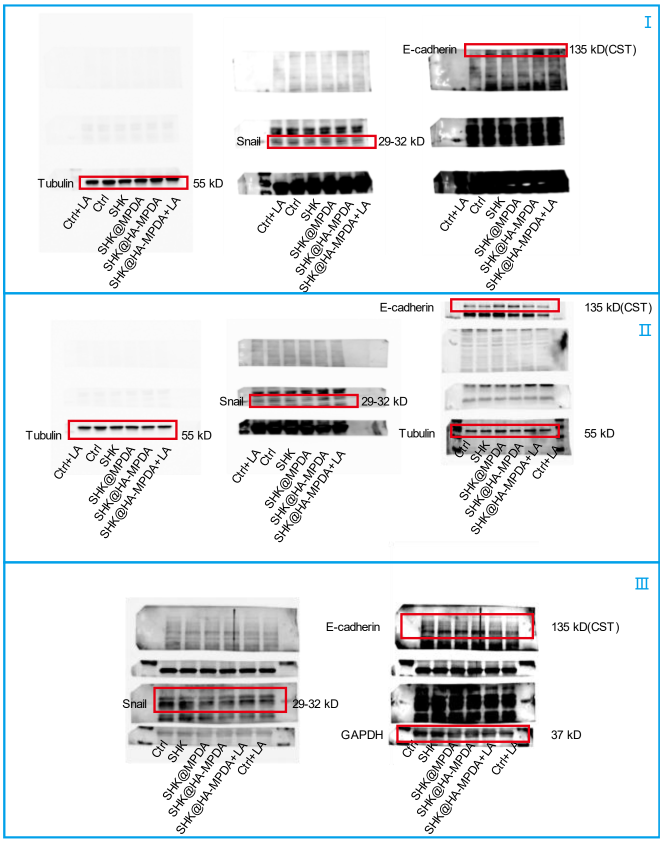

Appendix 8. The un-chopped images of Fig. 10D.

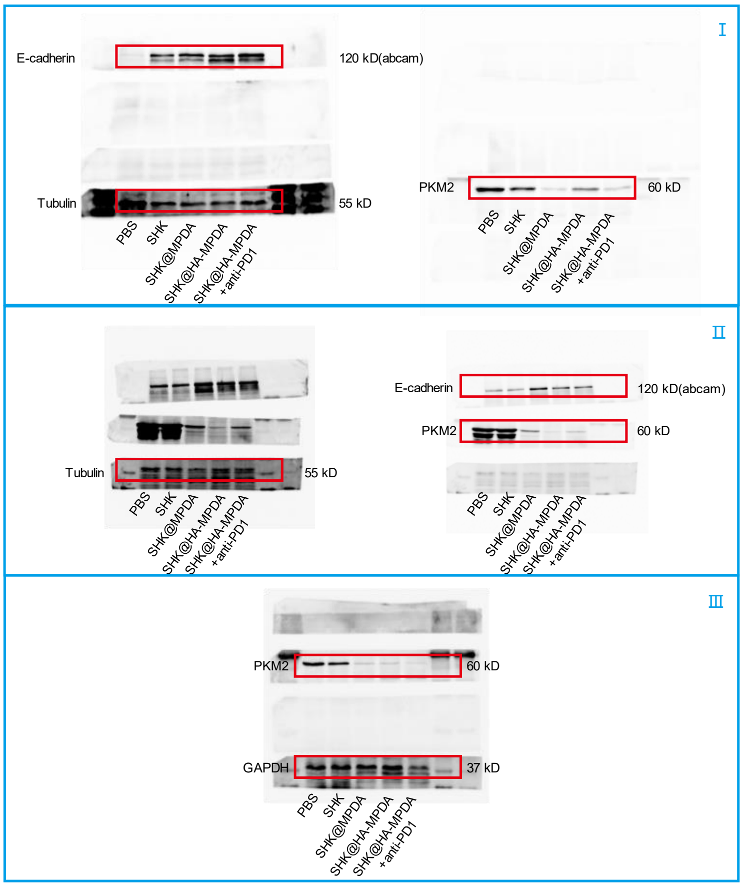

Appendix 9. The un-chopped images of Fig. S2A and Fig. S4A.

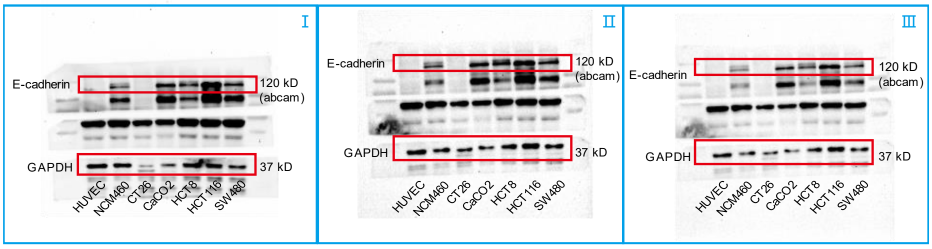

Appendix 10. The un-chopped images of Fig. S2C.

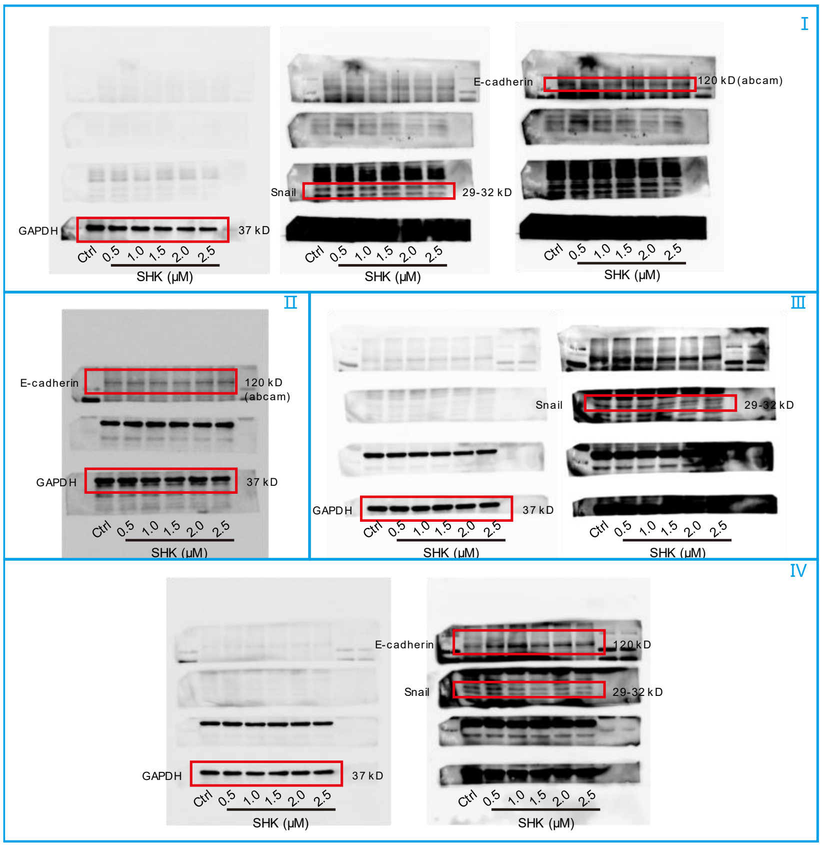

Appendix 11. The un-chopped images of Fig. S2G.

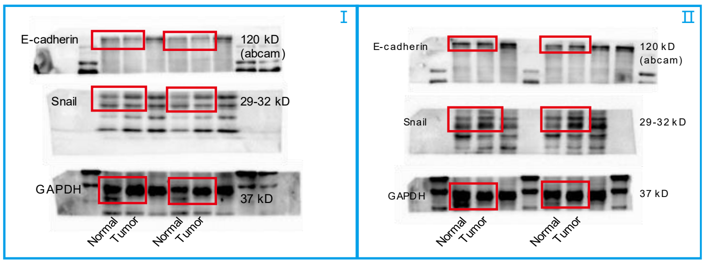

Appendix 12. The un-chopped images of Fig. S2I.

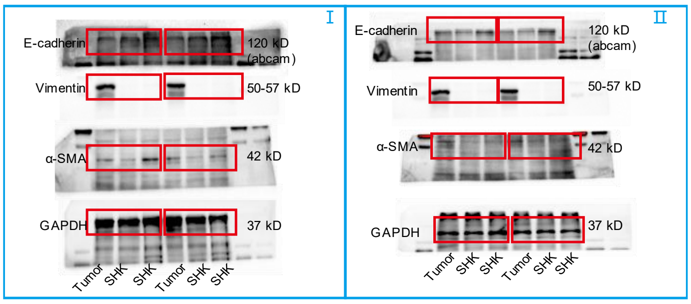

Appendix 13. The un-chopped images of Fig. S4C.

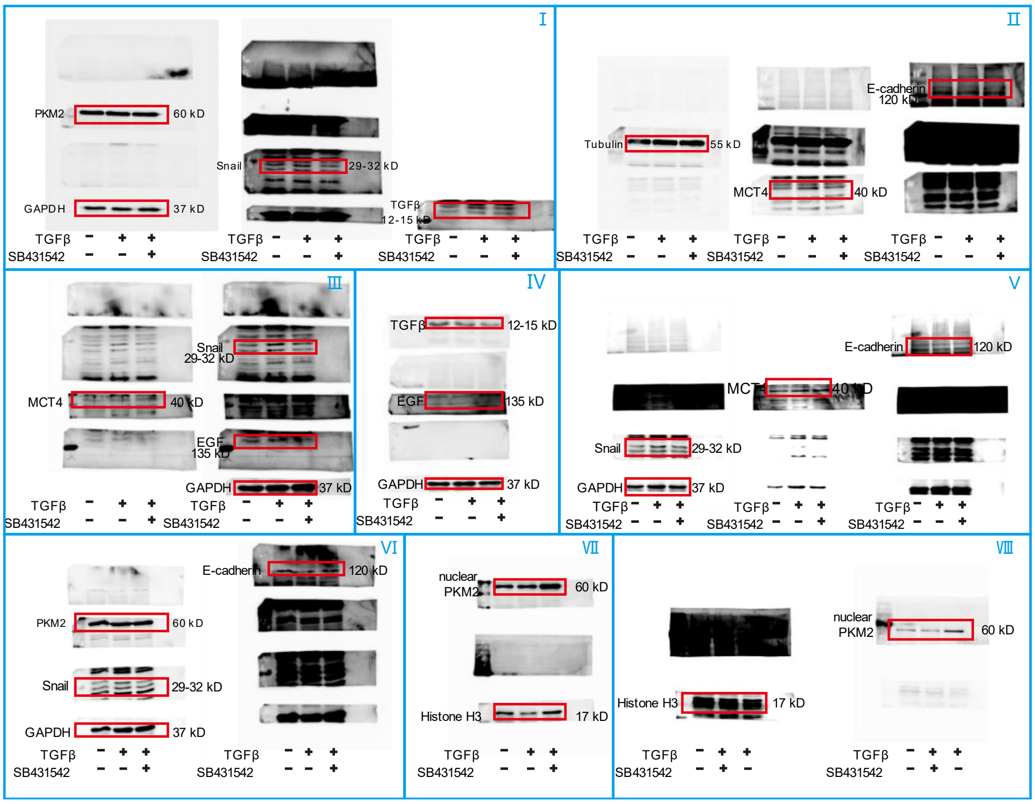

Appendix 14. The un-chopped images of Fig. S4F.

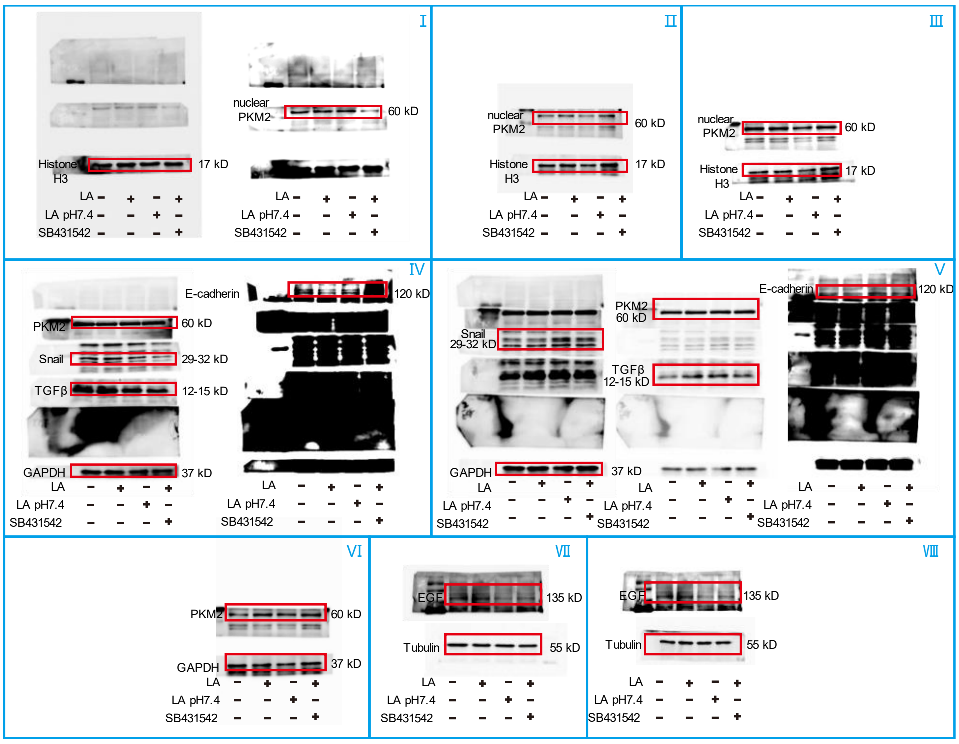

Appendix 15. The un-chopped images of Fig. S6A.

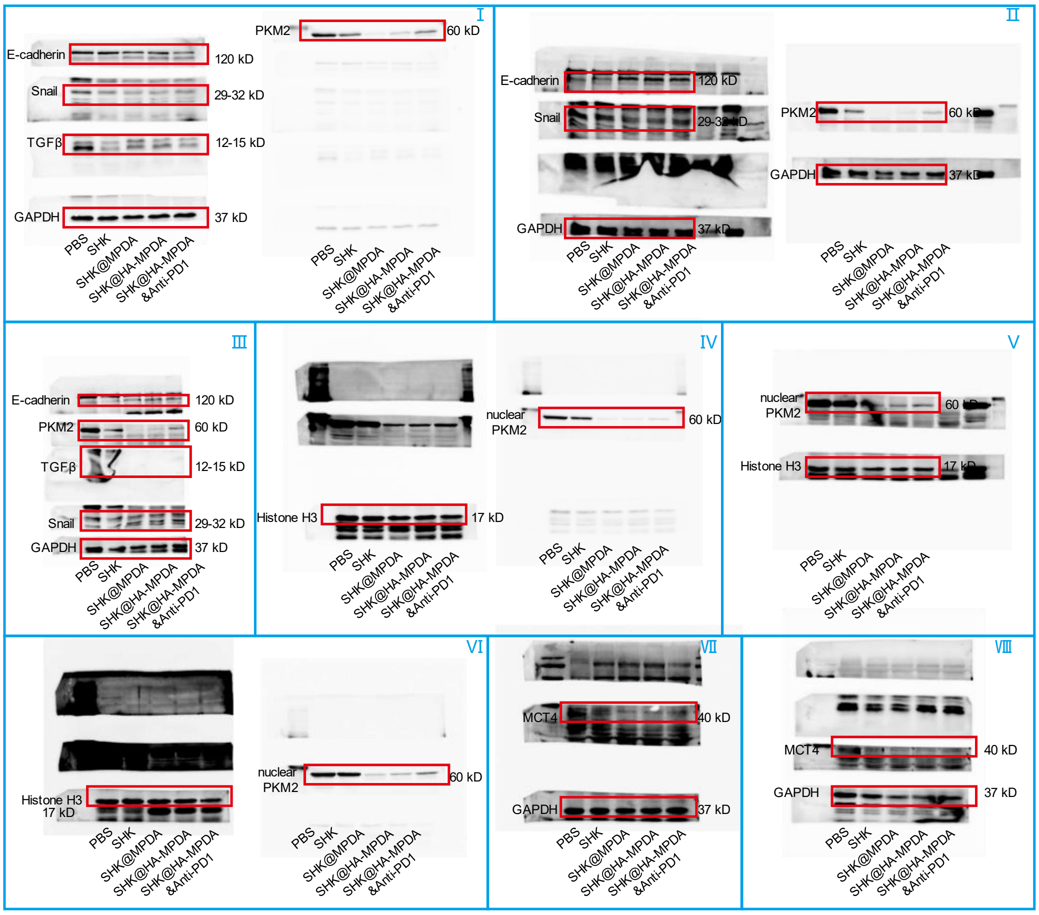

Supplement: Supplementary file 1 — Supplementary Material 1 [file 13046_2023_2688_MOESM1_ESM.pdf]
