# Supplementary material for: Regulating lactate-related immunometabolism and EMT reversal for colorectal cancer liver metastases using shikonin targeted delivery
Source: J Exp Clin Cancer Res. 2023 May 10;42:117. doi: 10.1186/s13046-023-02688-z (PMC10170793; doi:10.1186/s13046-023-02688-z)

**Fig. 1.** The upregulated expression of PKM2 in CRC and CRLM. ([A](#图1A)) Primary CRC patients overexpressed *pkm2* mRNA compared to normal tissue in the TCGA database. ([B](#图1B)) Patients with low *pkm2* expression in the TCGA database had significantly longer DSS (p = 0.01) than those with high *pkm2* expression. ([C](#图1C)) In the GSE21510 data set, the expression of *pkm2* mRNA of liver tissue of normal, CRC, and CRLM. ([D](#图1D)) and ([E](#图1E)) The expression and grayscale analysis of PKM2 in various colorectal cancer cells. ([F](#图1F)) Immunohistochemistry of PKM2 of liver tissue in normal or CRLM mice. CRC: Colorectal cancer cells; CRLM: Colorectal cancer liver metastasis. Data are presented as mean ± SD. **P*<0.05, ***P*<0.01, ****P*<0.001.


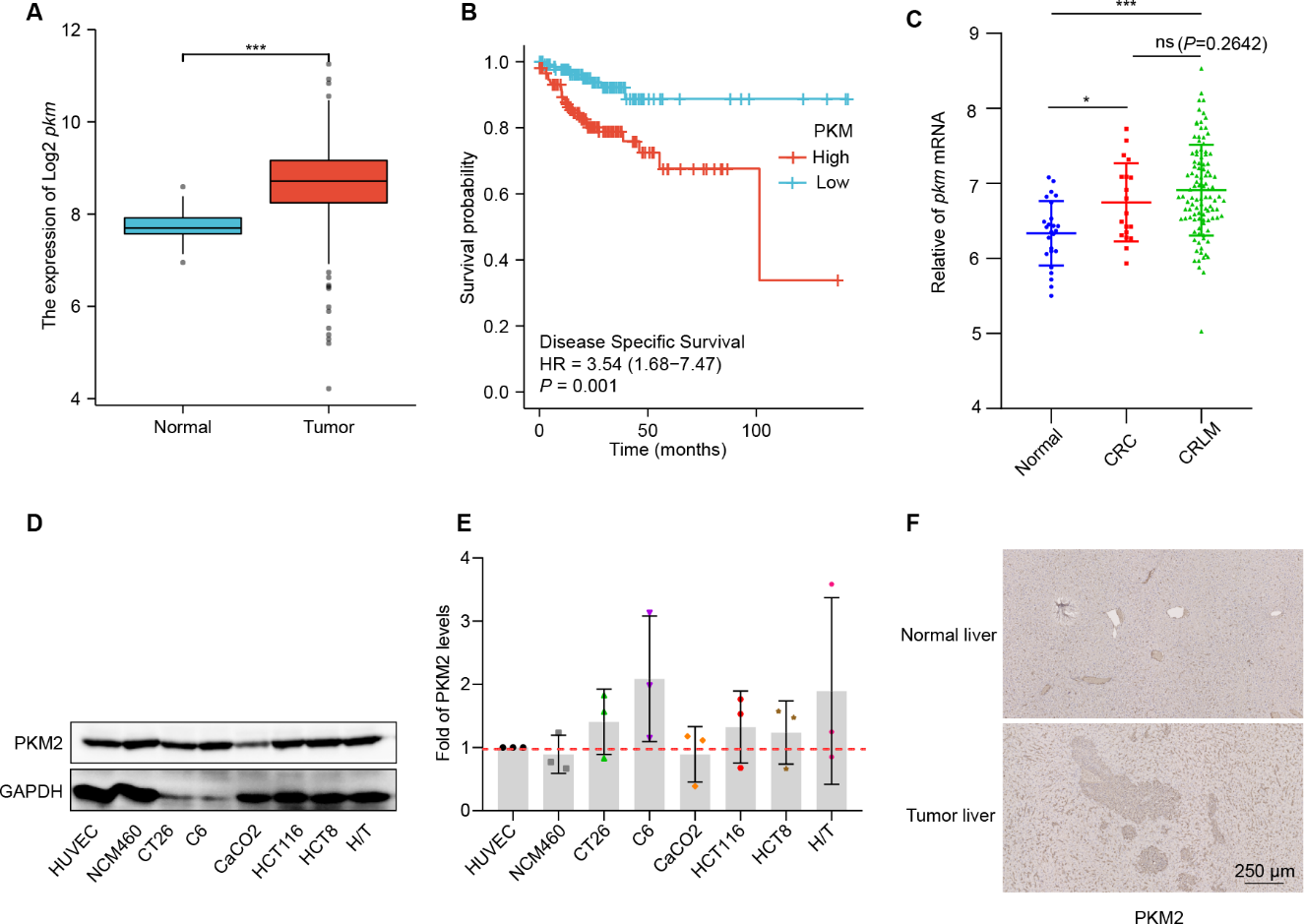


**Fig. 2.** Characterization of nanoparticles (n=3). ([A](#图2A)) Schematic illustration of SHK@HA-MPDA nanoparticles. The particle size distribution and TEM of MPDA, SHK@MPDA, and SHK@HA-MPDA ([B](#图2B), [C](#图2C), and [D](#图2D)). ([E](#图2E)) The zeta potential of nanoparticles. ([F](#图2F)) Nitrogen adsorption isotherms of nanoparticles. ([G](#图2G)) The pore size distribution from the corresponding desorption isotherm. ([H](#图2H)) The cumulative drug release of nanoparticles in PBS containing 0.5% SDS. ([I](#图2I)) The serum stability of nanoparticles in PBS containing 10% FBS.


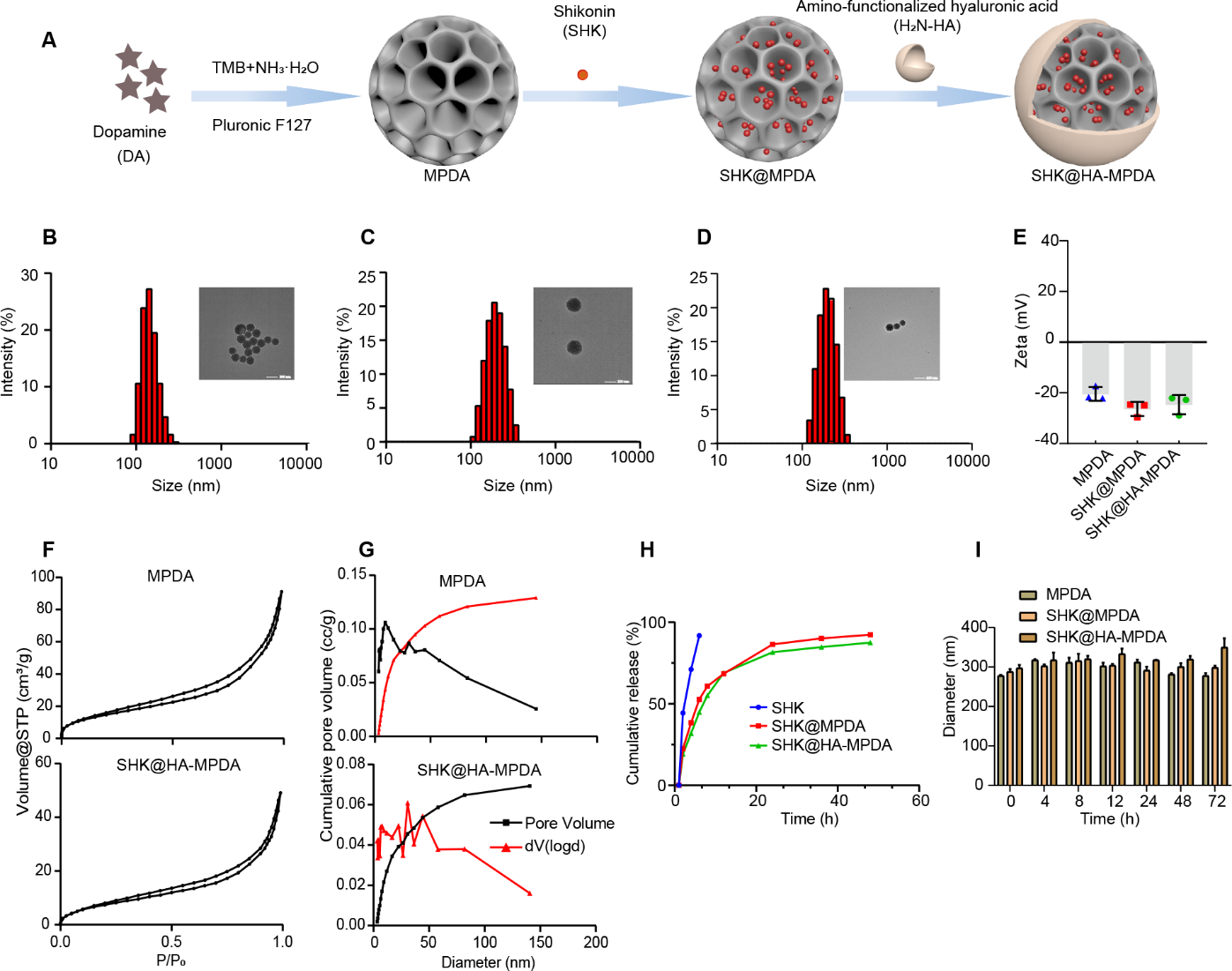


**Fig. 3**. Cellular uptake of nanoparticles. ([A](#图S3A)) and ([B](#图3B)) The expression and grayscale analysis of CD44 in NCM460, CT26, and CT26-Luc. ([C](#图3C)) The cellular uptake of different ratios of HA and MPDA in CT26 cells and the statistical analysis of cellular efficiency in ([D](#图3D)). ([E](#图3E)) The cell uptake of nanoparticles with or without HA in CT26 cells and the statistical analysis of cellular efficiency in ([F](#图3F)). ([G](#图3G)) The inverted microscope image of uptake of C6@HA-MPDA along with time in CT26 cells. Data are presented as mean ± SD (n=3). **P*<0.05, ***P*<0.01, ****P*<0.001.


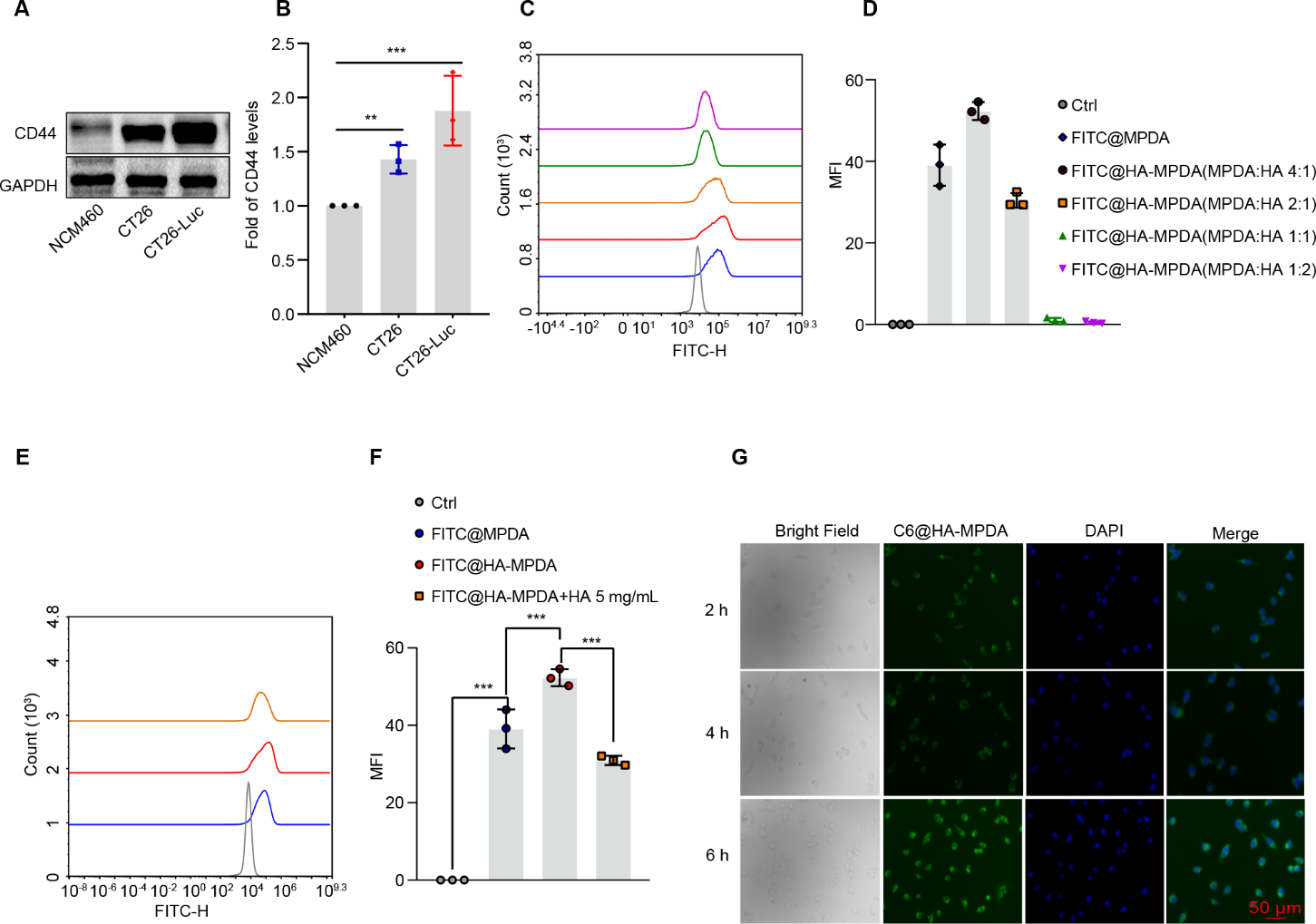


**Fig. 4.** The regulation glycolysis and cell proliferation of nanoparticles in CT26 cells. ([A](#图4A)) The cytotoxicity test of nanoparticles. ([B](#图4B)) The cytotoxicity test of SHK@HA-MPDA in NCM460 cells. ([C](#图4C)) The cytotoxicity test of MPDA in CT26 cells. ([D](#图4D)) and ([E](#图4E)) Quantitative of exocellular lactate and intracellular ATP of CT26 cells treated with nanoparticles. ([F](#图4F)) and ([G](#图4G))The expression and grayscale analysis of PKM2 and c-Myc in CT26 cells after treatment by nanoparticles. ([H](#图4H)) The Schematic mechanism of the nanoparticles regulating glycolysis and proliferation. Data are presented as mean ± SD (n=3). **P*<0.05, ***P*<0.01, ****P*<0.001.


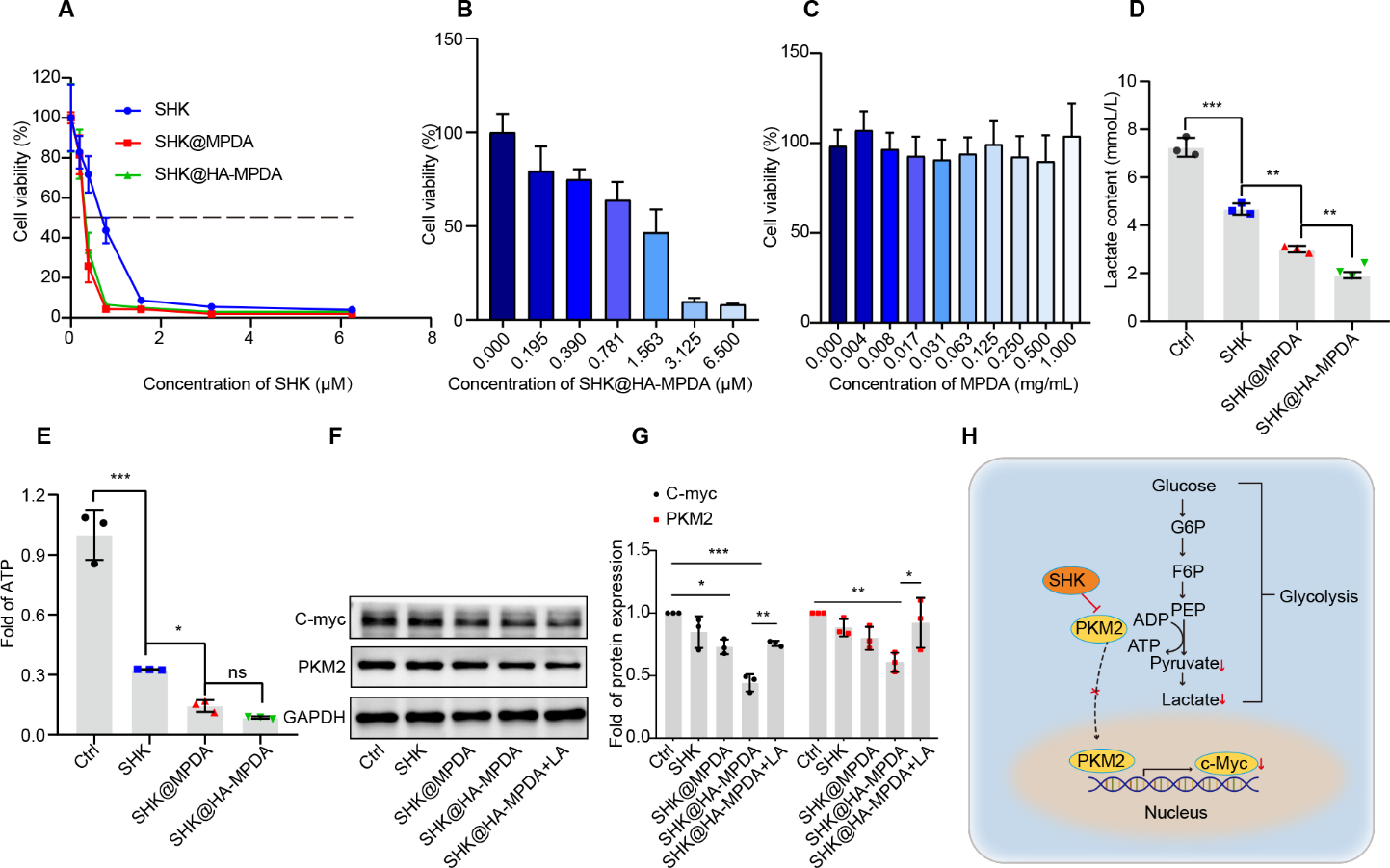


**Fig. 5.** Remodeling of immunometabolism. ([A](#图5A)) Analysis of calreticulin eversion in CT26 cells by flow cytometry. ([B](#图5B)) and ([C](#图5C)) Western blot analysis of the released HMGB-1 of CT26 cells and grayscale analysis of HMGB1. ([D](#图5D)) Schematic of the ICD stimulated maturation of BMDC. ([E](#图5E)) CD80^+^CD86^+^ DC subsets were stimulated by CT26 cells after treatment (LPS: 200 ng/mL; LA: lactate 5 mM). (F) Schematic of ICD activated T cell immunity. ([G–I](#图5G_I)) The CD8^+^T subsets (CD8^+^Ki67^+^) and the cytotoxic substances of CD8^+^T (CD8^+^IFNγ^+^, CD8^+^Granzyme B^+^) (Untreated: CT26 co-incubated with DC and spleen lymphocytes; LPS: 200 ng/mL; LA: 10 mM). Data are presented as mean ± SD (n=3). **P*<0.05, ***P*<0.01, ****P*<0.001.


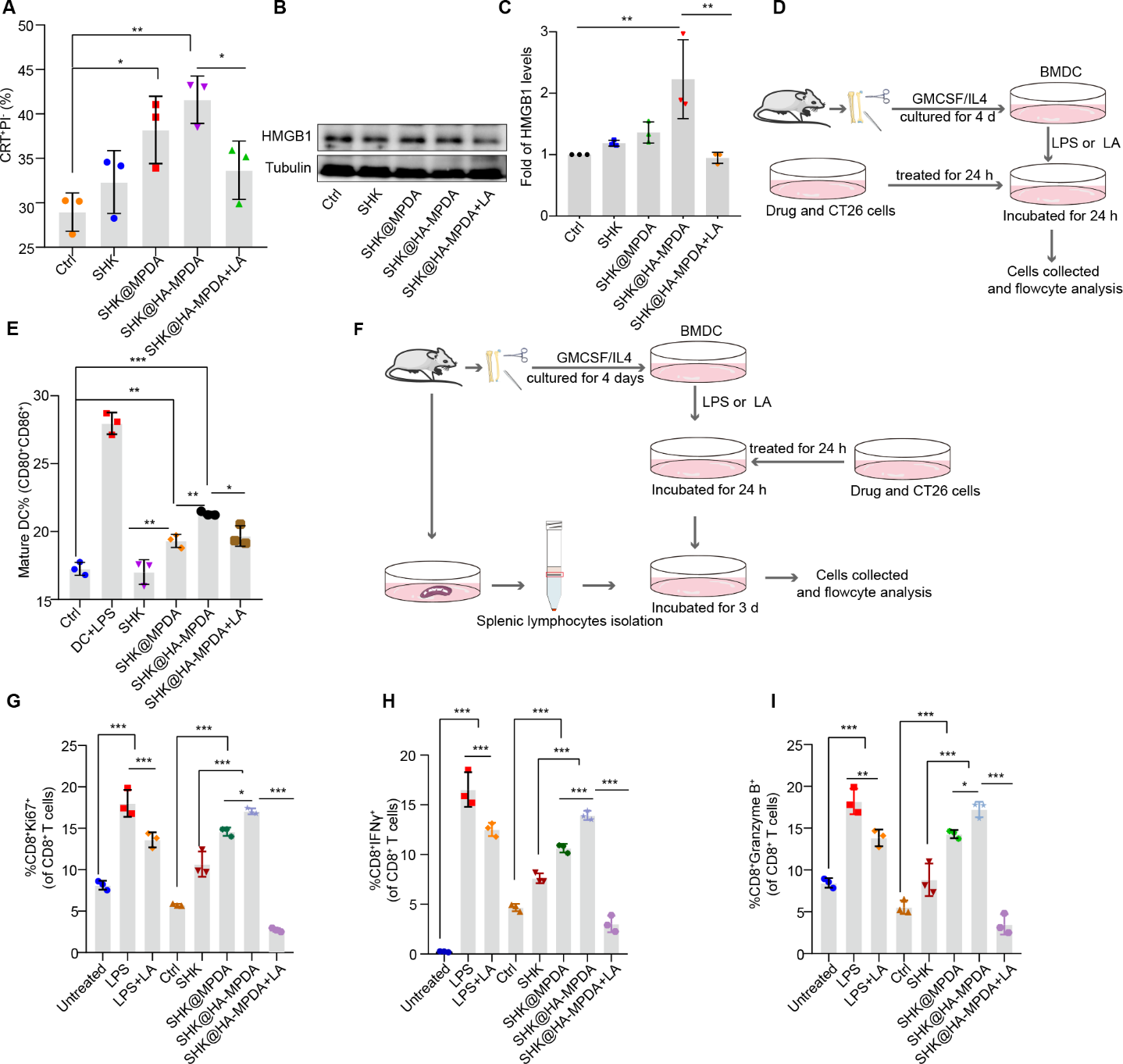


**Fig. 6.** The crosstalk between EMT and glycolysis. ([A](#图6A)) After treatment with nanoparticles, the representative images of colony-forming cells of CT26 cells and the statistical analysis of cell clusters in ([E](#图6E)). ([B](#图6B)) The representative images of scratch assay of CT26 cells and the statistical analysis of distance in ([F](#图6F)). The representative images of migration([C](#图6C)) and invasion ([D](#图6D)) of CT26 cells after suffering nanoparticles. ([G](#图6G)) Expression levels of epithelial or mesenchymal markers in CT26 cells were analyzed by western blotting. ([H](#图6H)) Expression levels of epithelial or mesenchymal markers of CT26 cells after suffering SHK@HA-MPDA but intervening with or without LA or SB431542 were analyzed by western blotting (LA: 10 mM; SB431542: 5 μM). ([I](#图6I)) The schematic of the mechanism between EMT and glycolysis in mesenchymal-like cells and epithelial-like cells. Data are presented as mean ± SD (n=3). **P*<0.05, ***P*<0.01, ****P*<0.001.


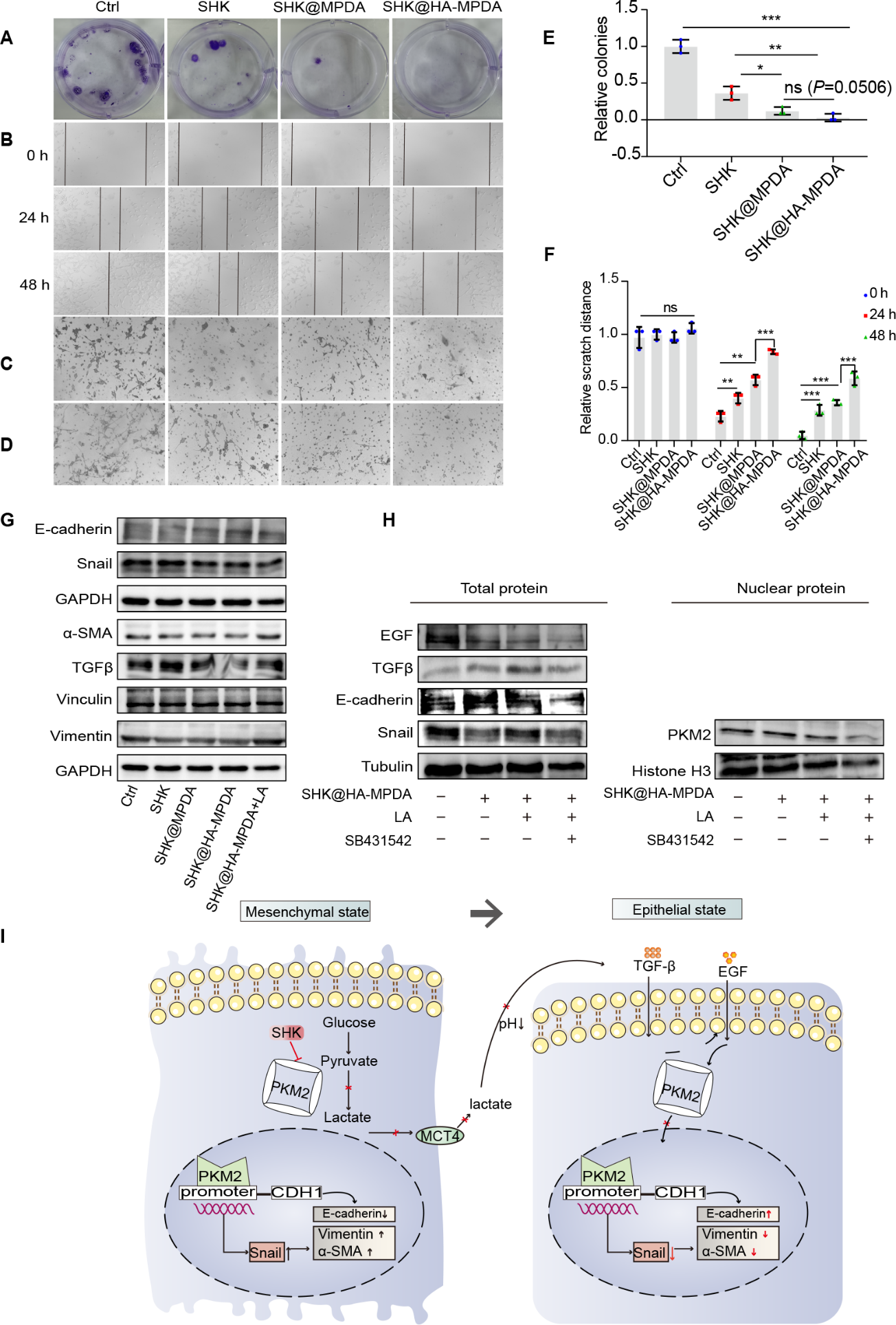


**Fig. 7.** Downstream migration of MDSCs in the early colorectal cancer reversed the EMT in epithelial-like cells in vitro. ([A](#图7A)) The schematic of MDSCs from the early colorectal cancer migrated to CT26 cells. ([B](#图7B)) The MDSCs of bone marrow (BM) or spleen (SL) from normal or tumor mice were analyzed by flow cytometry. The migration capability of MDSCs from BM ([C](#图7C)) or SL ([D](#图7D)) of tumor mice after treatment with nanoparticles in CT26 cells. ([E](#图7E)) LA intensified the migration capability of MDSCs from BM or SL of tumor mice in CT26 cells. ([F](#图7F)) SHK@HA-MPDA inhibited the migration of MDSCs but was blocked by LA (LA 10 mM). ([G](#图7G)) The schematic of MDSCs from tumor mice induced the epithelial-like cells EMT. ([H](#图7H)) and ([I](#图7I)) Western blot analysis and grayscale analysis of epithelial or mesenchymal markers in SW480 after co-incubating with a mix of MDSCs/CT26 cells. Data are presented as mean ± SD (n=3). **P*<0.05, ***P*<0.01, ****P*<0.001.


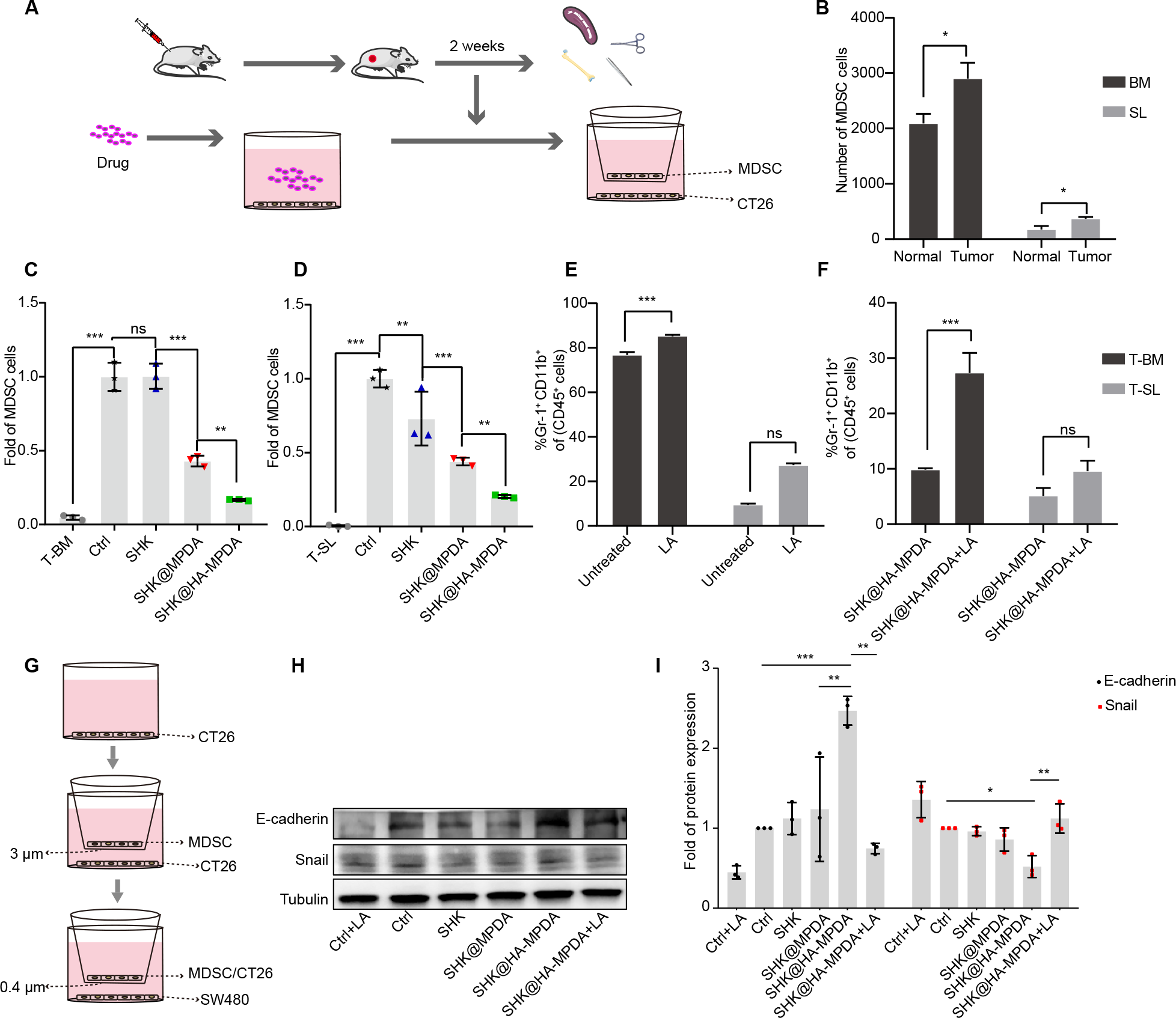


**Fig. 8.** In vivo therapeutic efficacy of nanoparticles in a mouse model with early liver metastasis of colorectal cancer. ([A](#图8A)) Therapeutic schedule. ([B](#图8B)) The image of liver nodules of groups and the statistical analysis in ([C](#图8C)). ([D](#图8D)) The H&E strain of liver tissues. ([E–F](#图8E)) Liver function index of serum (AST, ALT, and ALP). Data are presented as mean ± SD (n=5). **P*<0.05, ***P*<0.01, ****P*<0.001.


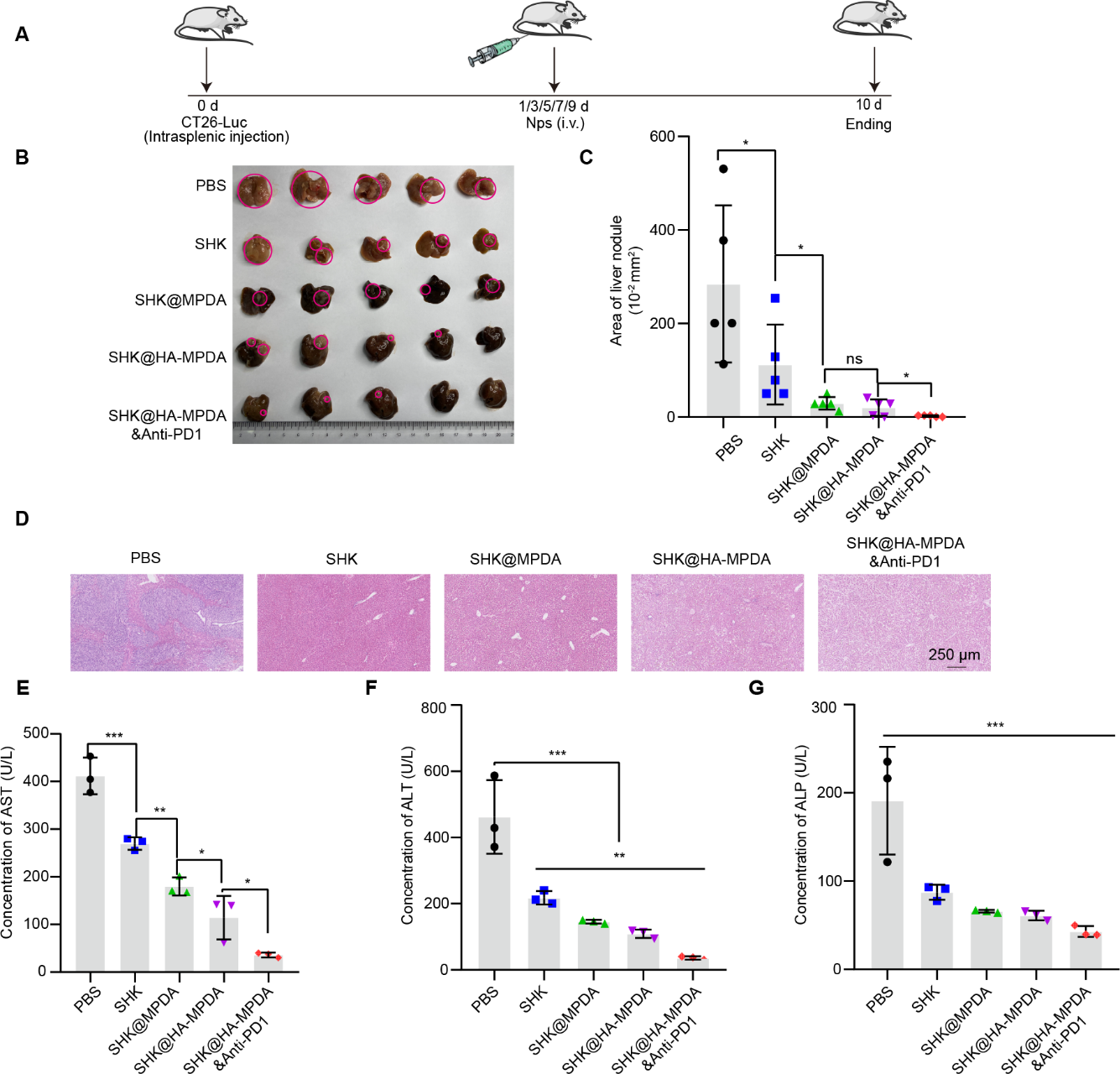


**Fig. 9.** In vivo therapeutic efficacy of a mouse model with advanced liver metastasis of colorectal cancer and subcutaneous recurrence. ([A](#图9A)) Therapeutic schedule. ([B](#图9B)) The image of liver nodules of groups and the statistical analysis in ([C](#图9C)). ([D](#图9D)) The H&E strain and immunohistochemistry of PKM2, E-cadherin, CD31, and VEGF in liver tissues. Data are presented as mean ± SD (n=4). **P*<0.05, ***P*<0.01, ****P*<0.001.


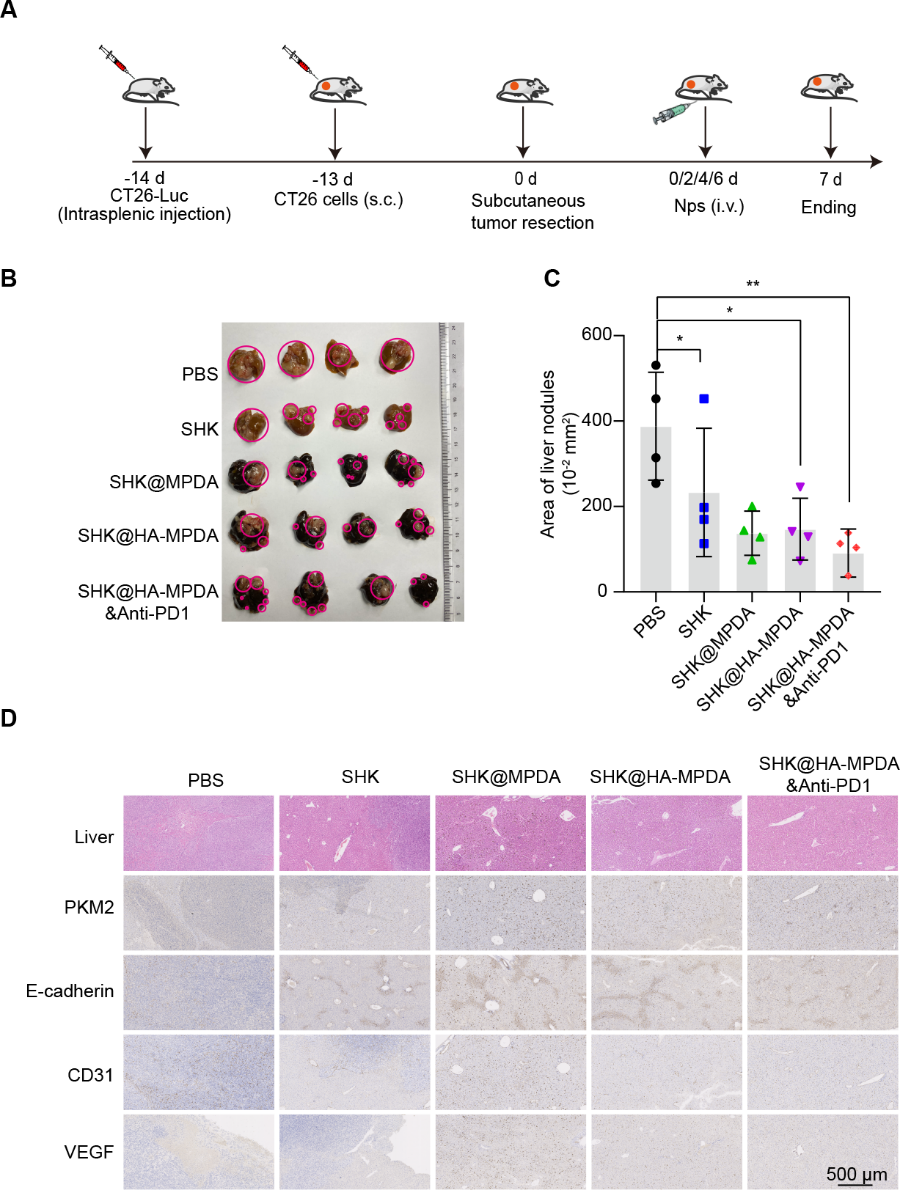


**[Fig. 10](#图10).** In vivo therapeutic efficacy of CT26 subcutaneous tumor in situ recurrence model. ([A](#图10A)) The image of tumor volume of groups. ([B](#图10B)) The tumor growth curve. ([C](#图10C)) Statistical analysis of tumor weight after surgical of primary tumors. ([D](#图10D)) and ([E](#图10E)) The expression and grayscale analysis of PKM2 of tumor tissues. ([F](#图10F)) The level of lactate in tumor tissues. The relative fold change of MDSCs (Gr-1^+^) ([G](#图10G)), NK (NK1.1^+^CD49b^+^) ([H](#图10H)), the CT8^+^T cells (CD8^+^Ki67^+^) ([I](#图10I)), and cytotoxicity CD8^+^ T cells (CD8^+^Granzyme B^+^ and CD8^+^IFNγ^+^) ([J and K](#图10J_k)). Data are presented as mean ± SD (n=4). **P*<0.05, ***P*<0.01, ****P*<0.001.


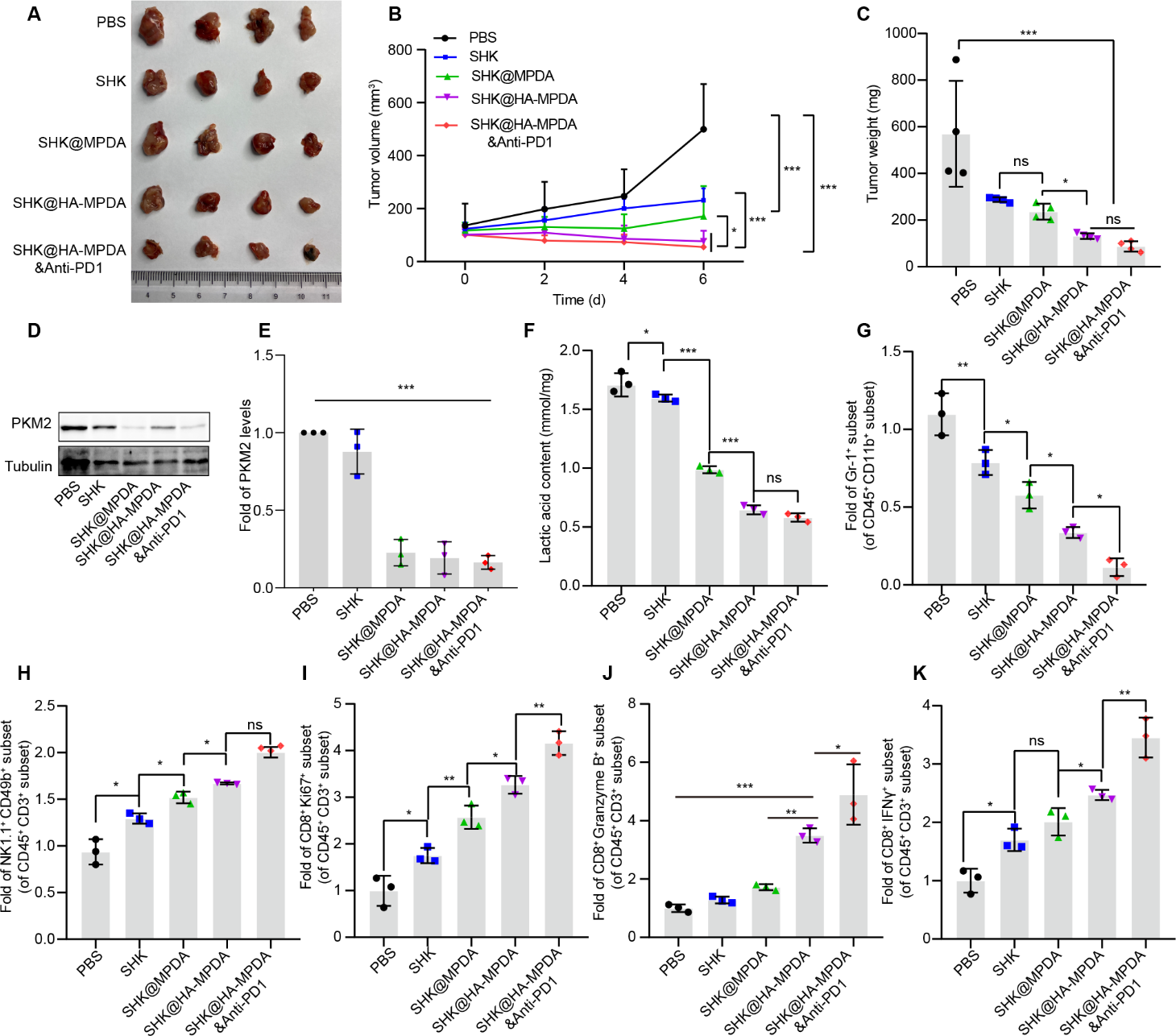


**Supporting Information**

**Regulating lactate-related immunometabolism and EMT reversal for colorectal cancer liver metastases using shikonin targeted delivery**

**[Fig. S1](#图S11)** The confocal images of CT26 cells and NCM460 cells.


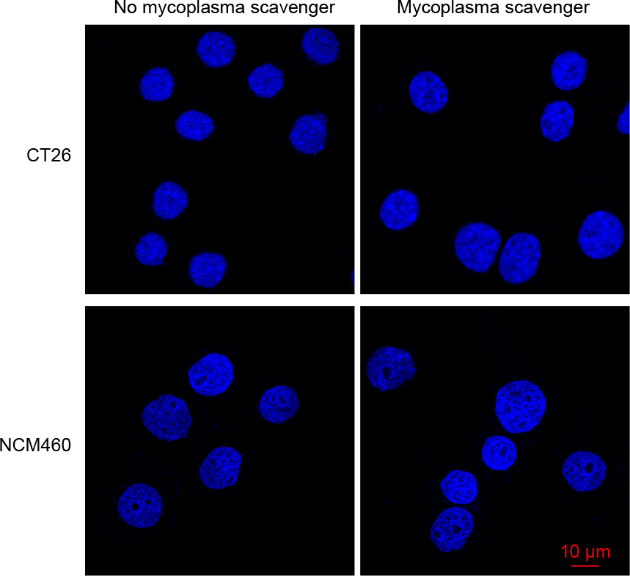


**Fig. S2.** Preliminary evaluation regulating EMT of SHK in a CRLM model. ([A](#图S2A)) and ([B](#图S2B)) The expression and grayscale analysis of E-cadherin of epithelial or mesenchymal markers in CT26 and NCM460 cells. ([C](#图S2C)) Expression of epithelial or mesenchymal markers in CT26 cells after treatment with free SHK. ([D](#图S2D)) The grayscale analysis of ([C](#图S2C)). ([E](#图S2E)) Therapeutic schedule. ([F](#图S2F)) The expression of E-cadherin of liver tissues in normal and tumor mice. ([G](#图S2G)) and ([H](#图S1H)) Expression and grayscale analysis of epithelial or mesenchymal markers of normal or tumor mice. ([I](#图S1I)) and ([J](#图S1J)) Western blot analyzed free SHK-regulated EMT in a CRLM model and grayscale analysis of EMT-related makers. ([K](#图S1K)) Grayscale analysis of Fig. 6[G](#图6G). ([L](#图S1L)) Grayscale analysis of Fig. 6[H](#图6H).


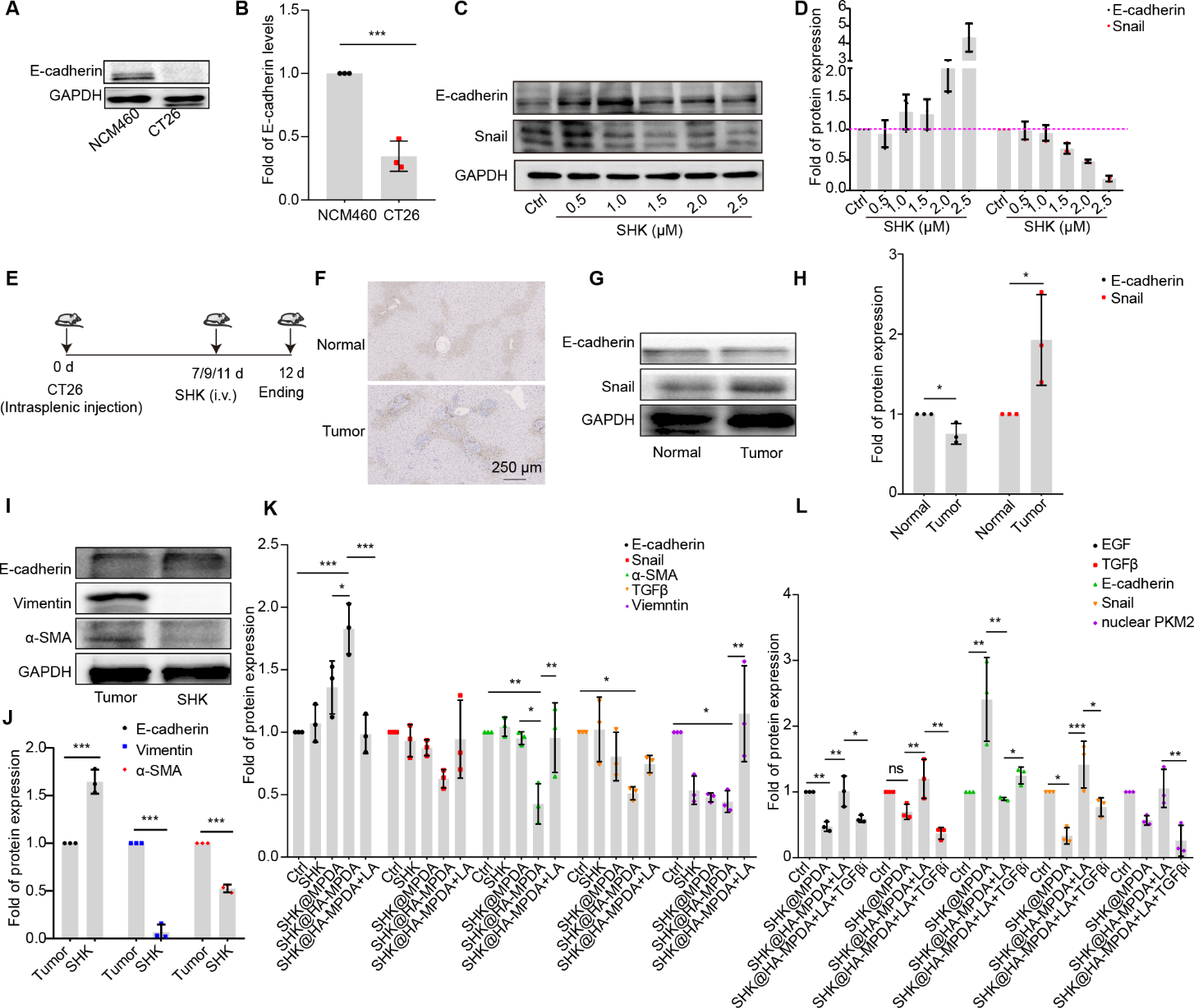


**Fig. S3.** Flow cytometry gate diagram of CTLs ([A](#图S1A)) and MDSCs ([B](#图S1B)) in vitro.


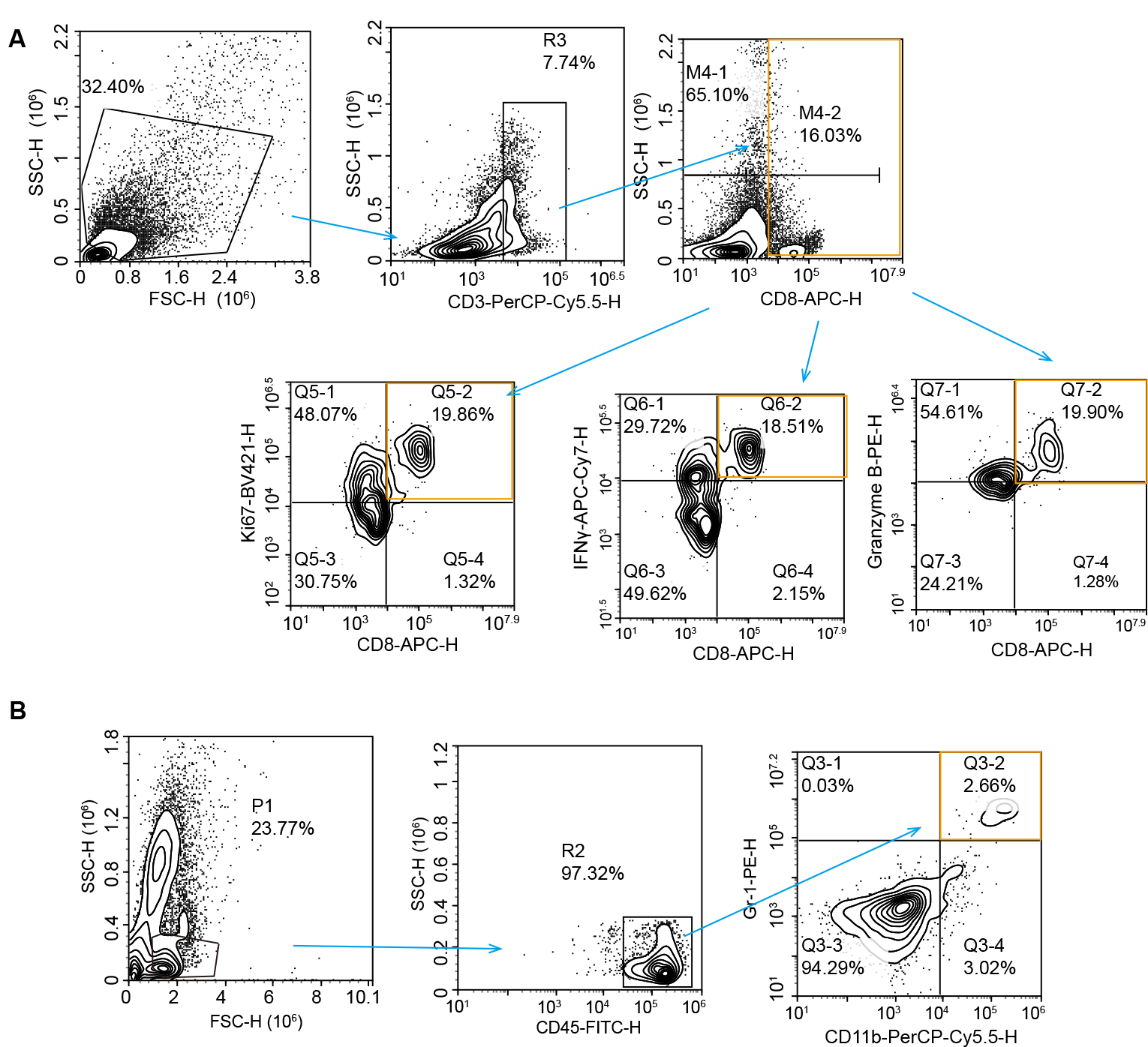


**Fig. S4.** LA inducing EMT depending on TGF/EGF in an EMT model of SW480 cells. ([A](#图S3A)) and ([B](#图S3B)) Expression and grayscale analysis of E-cadherin in colorectal cancer cells. ([C](#图S3C)) and ([D](#图S3D)) Western blot analysis of epithelial or mesenchymal markers in mesenchymal-like cells with or without TGFβ (10 ng/mL) and SB431542 (5 μM). ([E](#图S3E)) The LA production of mesenchymal-like cells and epithelial-like cells (TGFβ: 10 ng/mL). ([F](#图S3F)) and Western blot analysis of LA depended on pH-stimulated TGF/EGF inducing EMT in SW480 cells (LA: 20 mM; SB431542: 5 μM). ([G](#图S3G)) Grayscale analysis of ([F](#图S3F)).


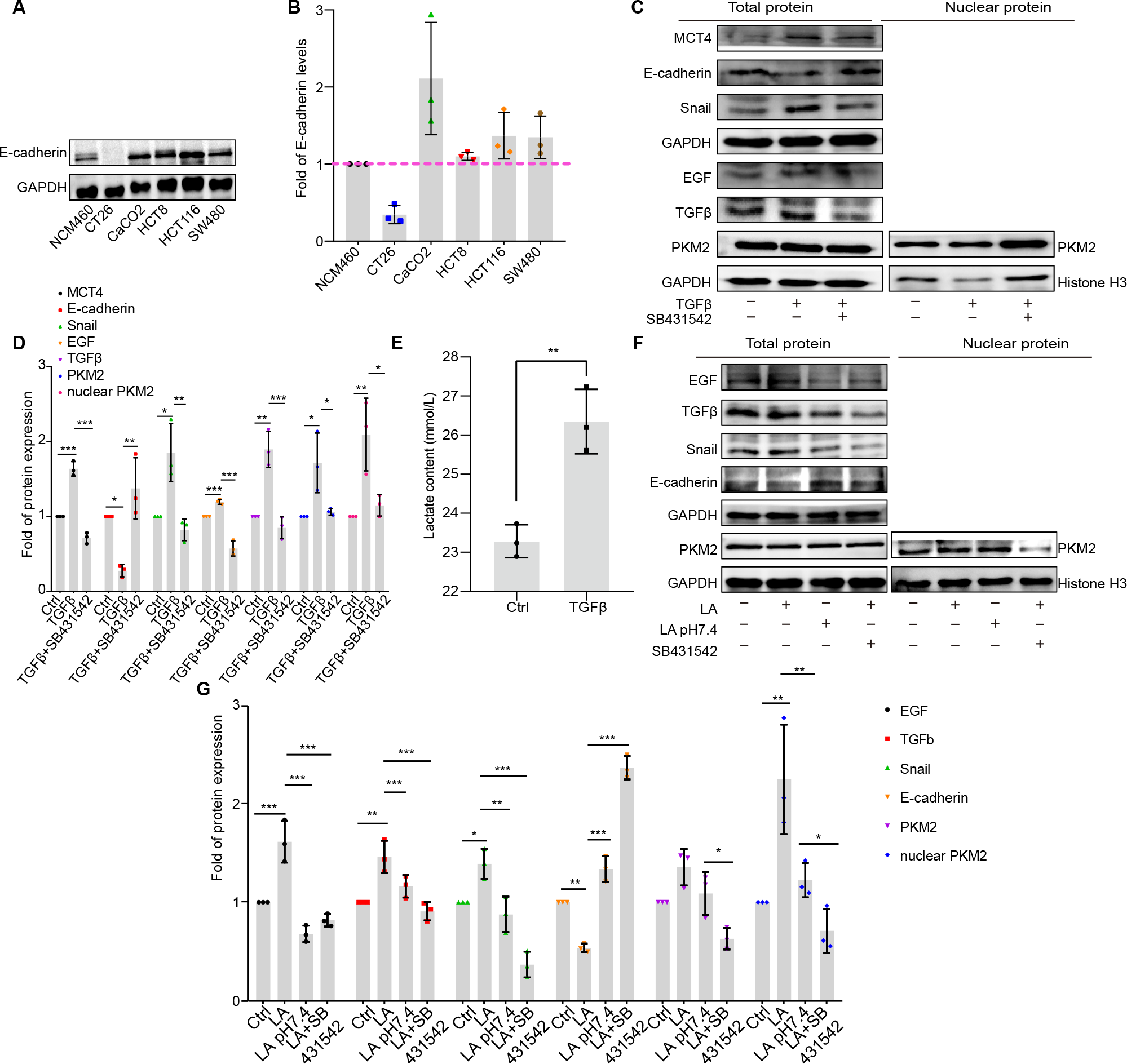


**Fig. S5.** The profiles of SHK@MPDA@HA nanosystem. ([A–D](#图S4A_C)) The photothermal performance of MPDA in vitro. The temperature changes curve with 808 NIR irradiation of CT26 subcutaneous tumor after treatment with SHK@MPDA ([E](#图S4E_F)) and SHK@MPDA@HA ([F](#图S4E_F)). The temperature changes curve ([G](#图S4G_H)) and image ([H](#图S4G_H)) of CT26 subcutaneous tumor with 808 NIR irradiation at 30 h.


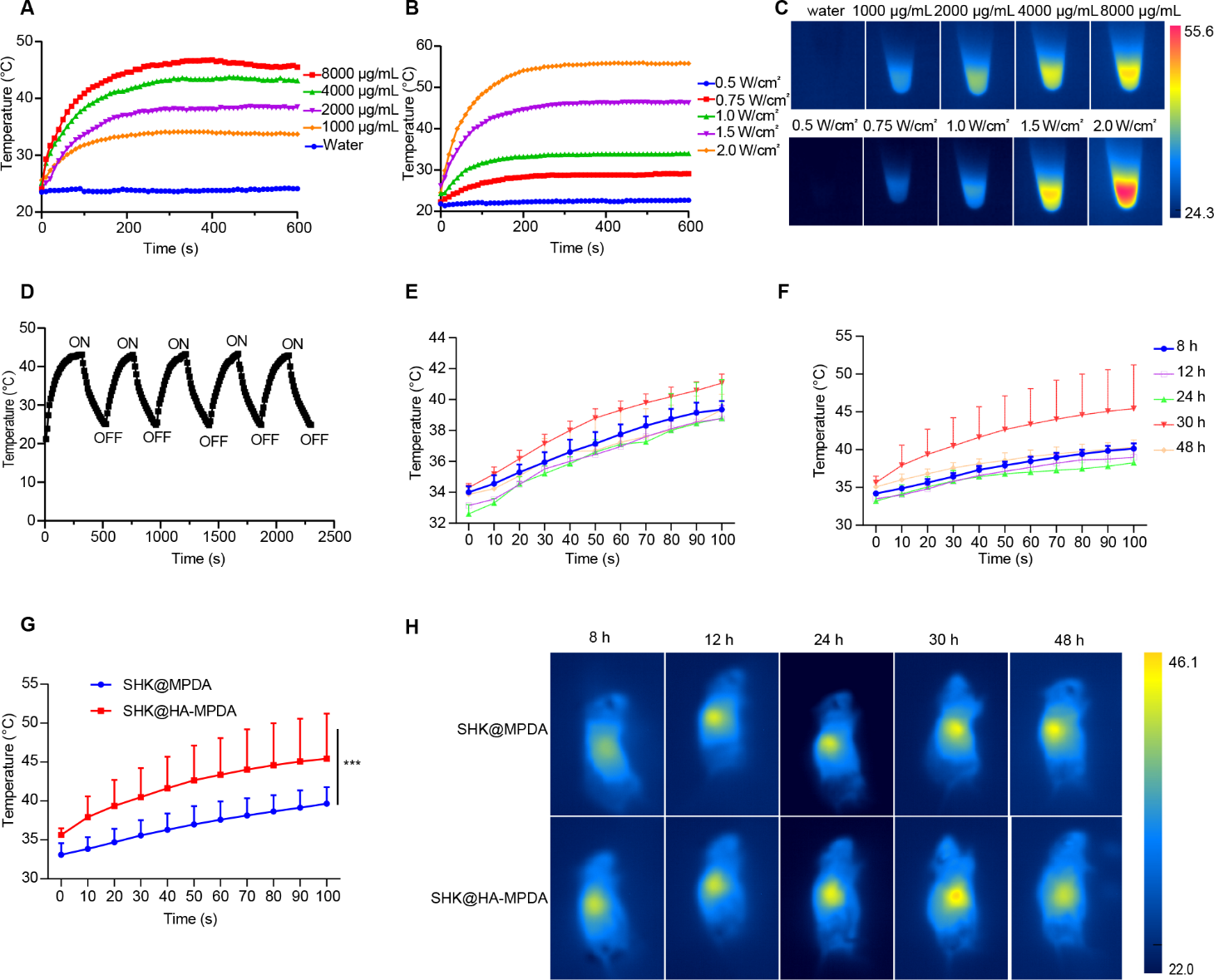


**[Fig. S6](#图S5)**. Reversion of EMT and remodeling tumor immunodepression microenvironment in early liver metastasis of colorectal cancer. ([A](#图S5A)) Western blot analysis of epithelial or mesenchymal markers of liver tissues. ([B](#图S5B)) Grayscale analysis of ([A](#图S5A)). [(C)](#图S5C) The relative fold change of the MDSCs subset (CD11b^+^Gr-1^+^). [(](#图S5D)[D)](#图S5D) and ([E](#图S5E))The relative fold change of the NK subset (CD49b^+^NK1.1^+^, CD49b^+^NK1.1^+^IFNγ^+^). ([F](#图S5F)) The relative fold change of the CT8^+^T cells (CD8^+^Ki67^+^) and ([G](#图S5G) and [H](#图S5H)) cytotoxicity CD8^+^ T cells (CD8^+^Granzyme B^+^ and CD8^+^IFNγ^+^)


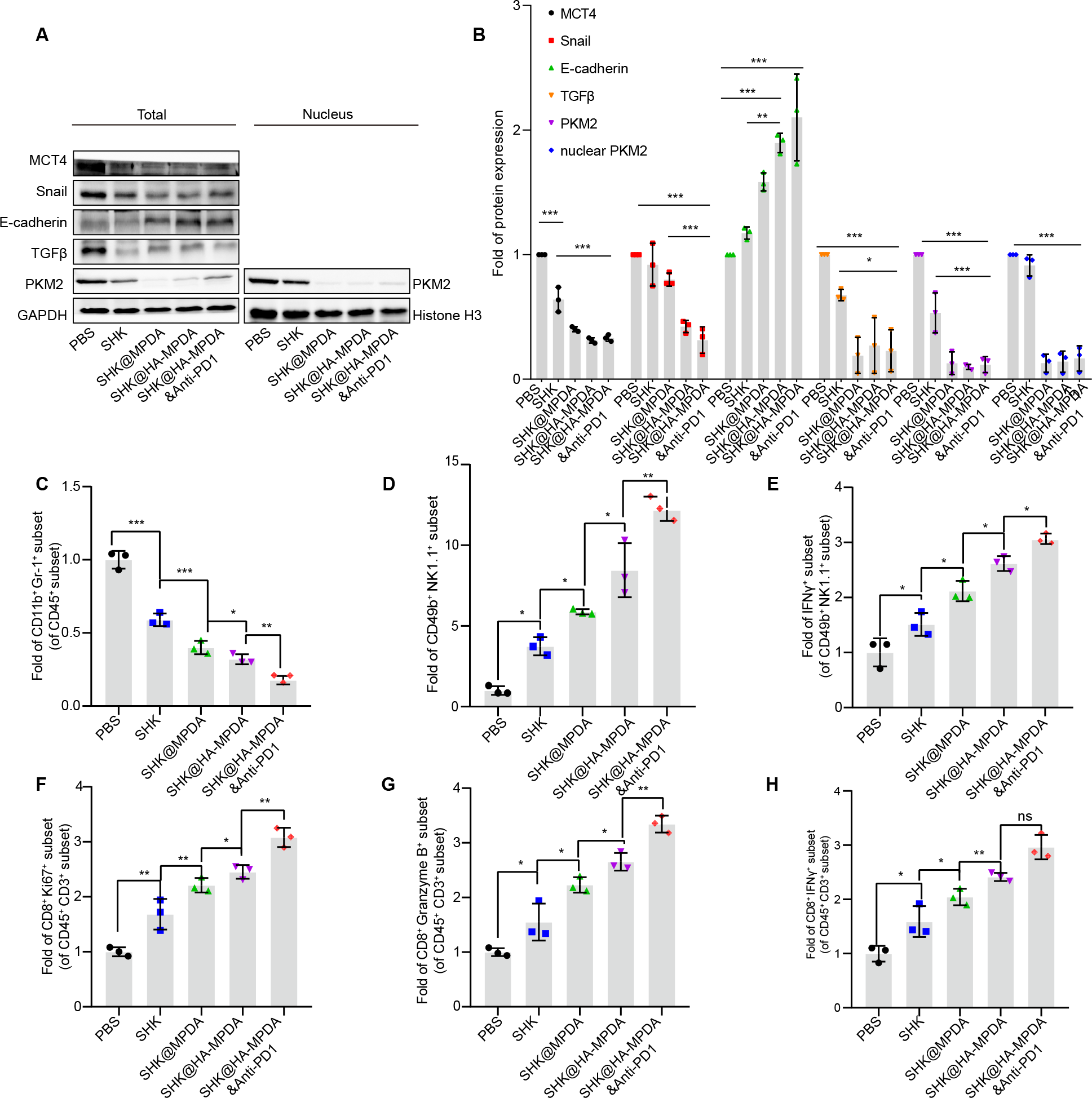


**[Fig. S7](#图S6).** Flow cytometry gate diagram of MDSCs (A), NK cells (B), and CTLs (C) in a mouse model with early liver metastasis of colorectal cancer.


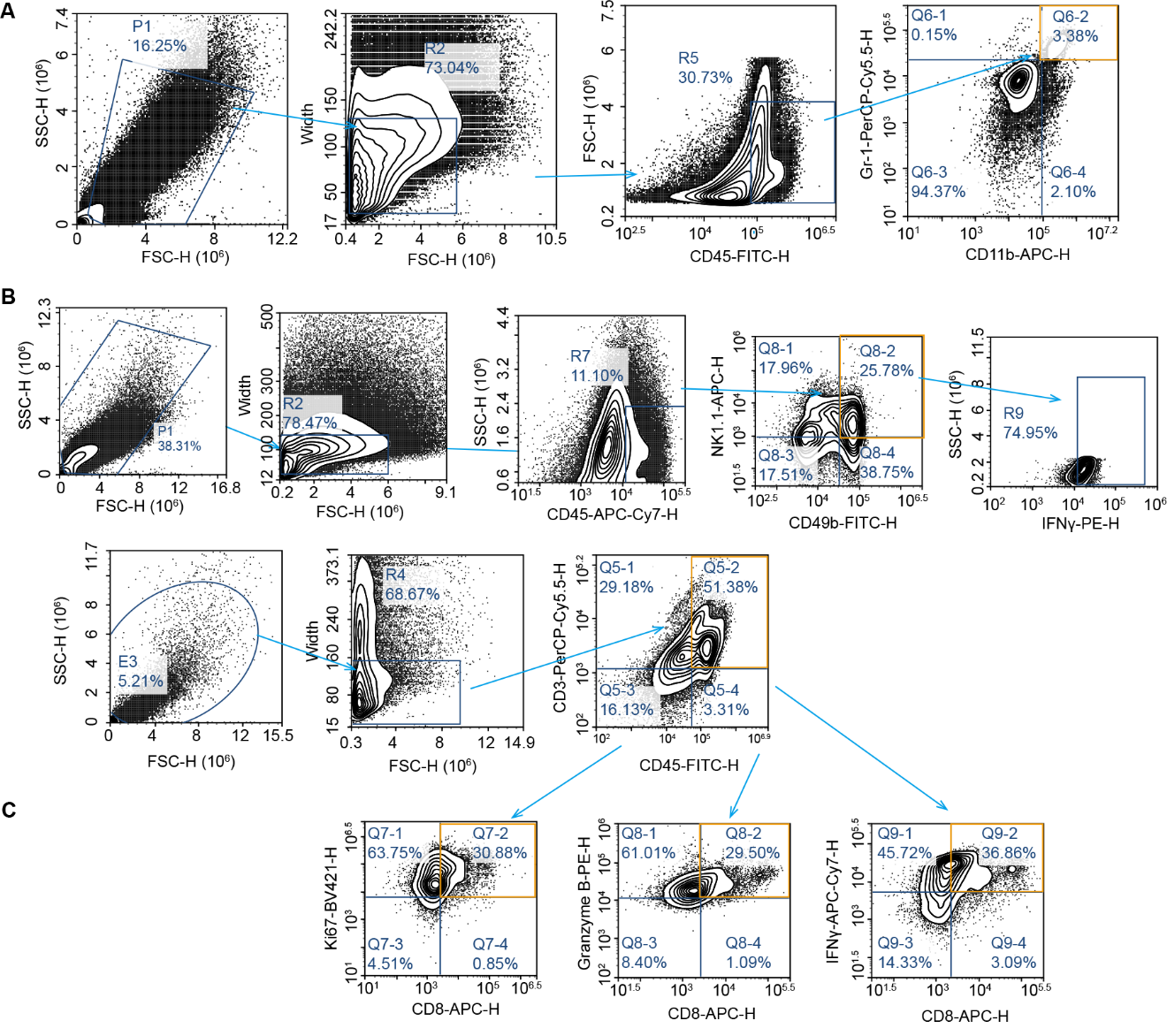


**[Fig. S8](#图S88)** The isolated tumor volume in a subcutaneous excision model at end point of experiment.


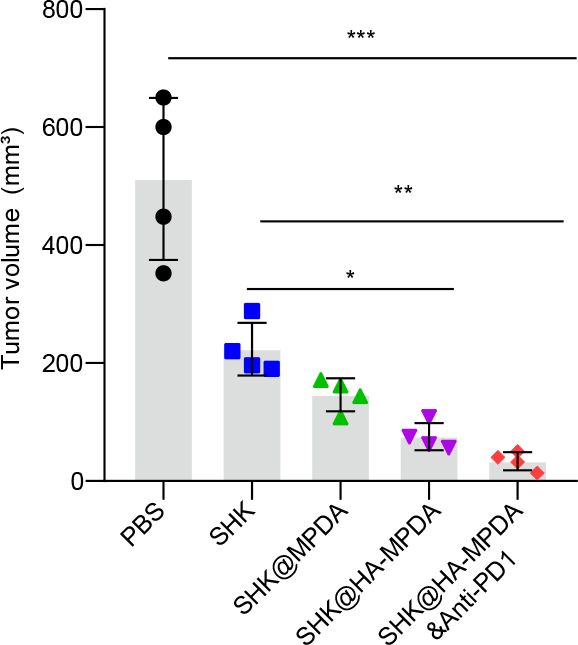


[**Fig. S9.**](#图S7) Flow cytometry gate diagram of MDSCs (A), NK cells (B), and CTLs (C) of CT26 subcutaneous tumor in situ recurrence model.


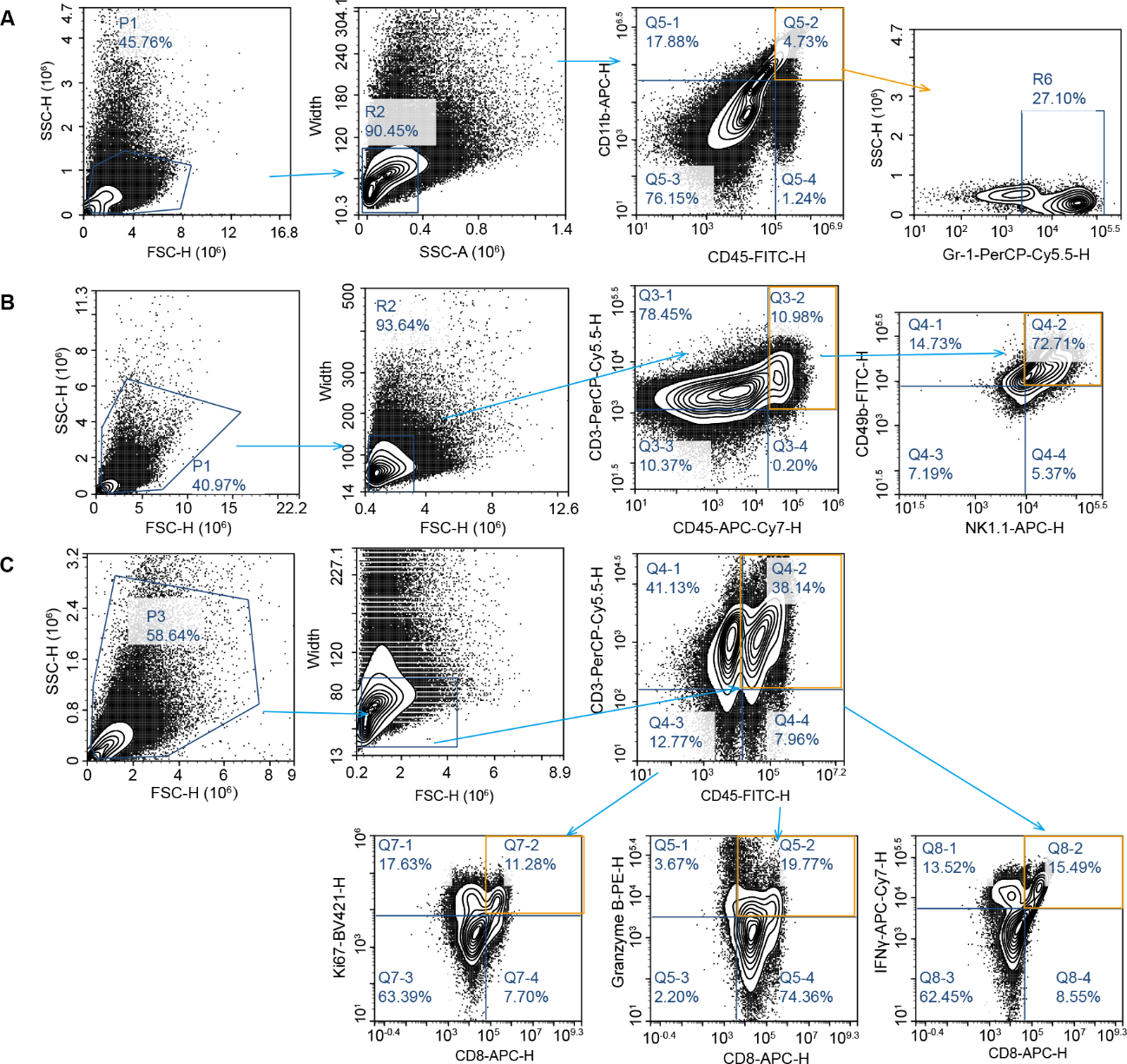


**[Fig. S10](#图S100b_J)** In vivo therapeutic efficacy of CT26 subcutaneous excision model. ([A](#图S100A)) Therapeutic schedule. (B) The tumor growth curve. (C) Statistical analysis of tumor weight after surgical of primary tumors. (D) The image of tumor volume of groups. (E) Statistical analysis of the isolated tumor volume of subcutaneous excision model at end point of experiment. (F–J) Individual tumor growth curves of PBS, MPDA, Anti-PD1, and SHK@HA-MPDA, and SHK@HA-MPDA&Anti-PD1 groups.


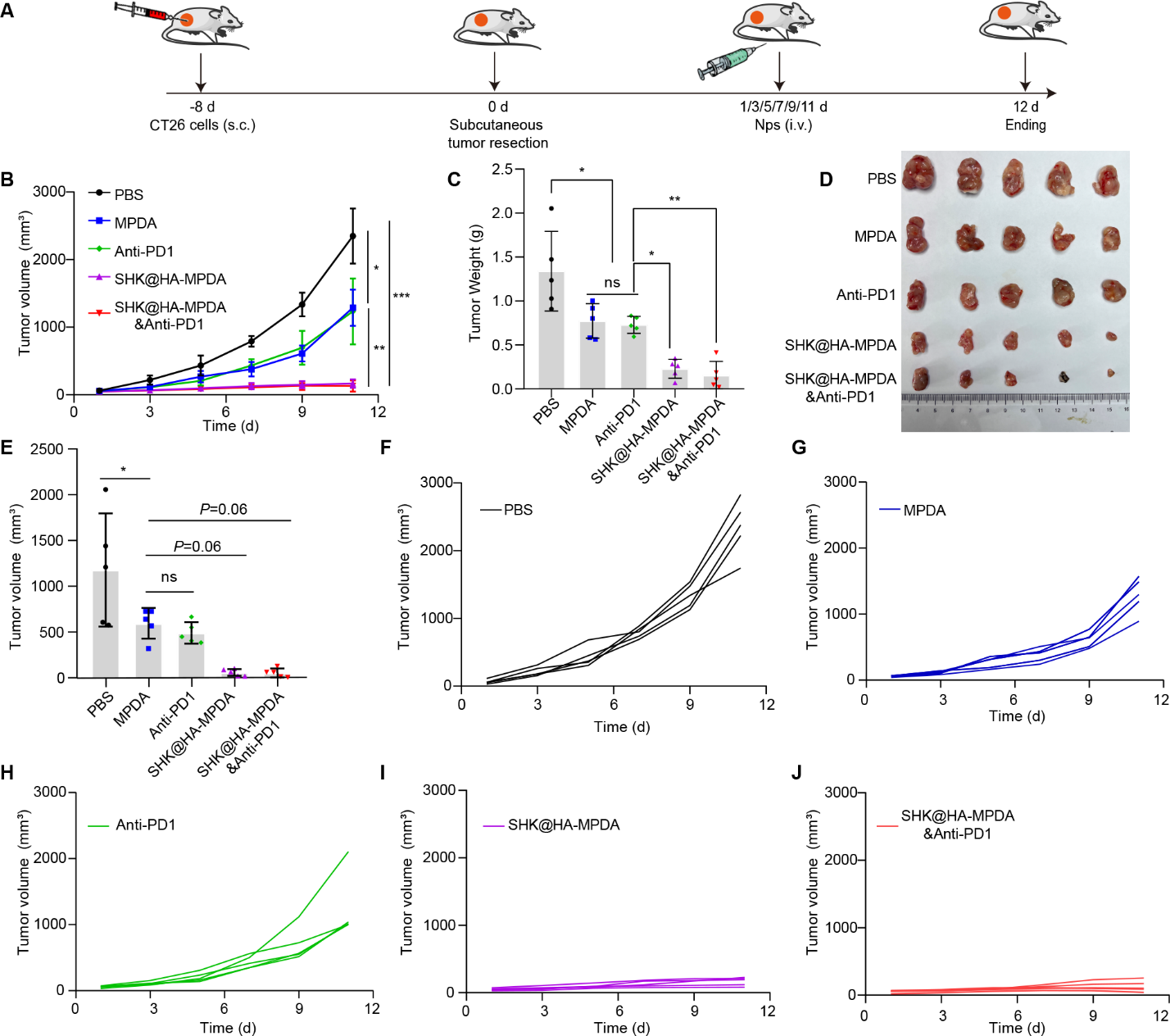


**[Fig. S11](#图S8).** (A–C) Preliminary safety evaluation.


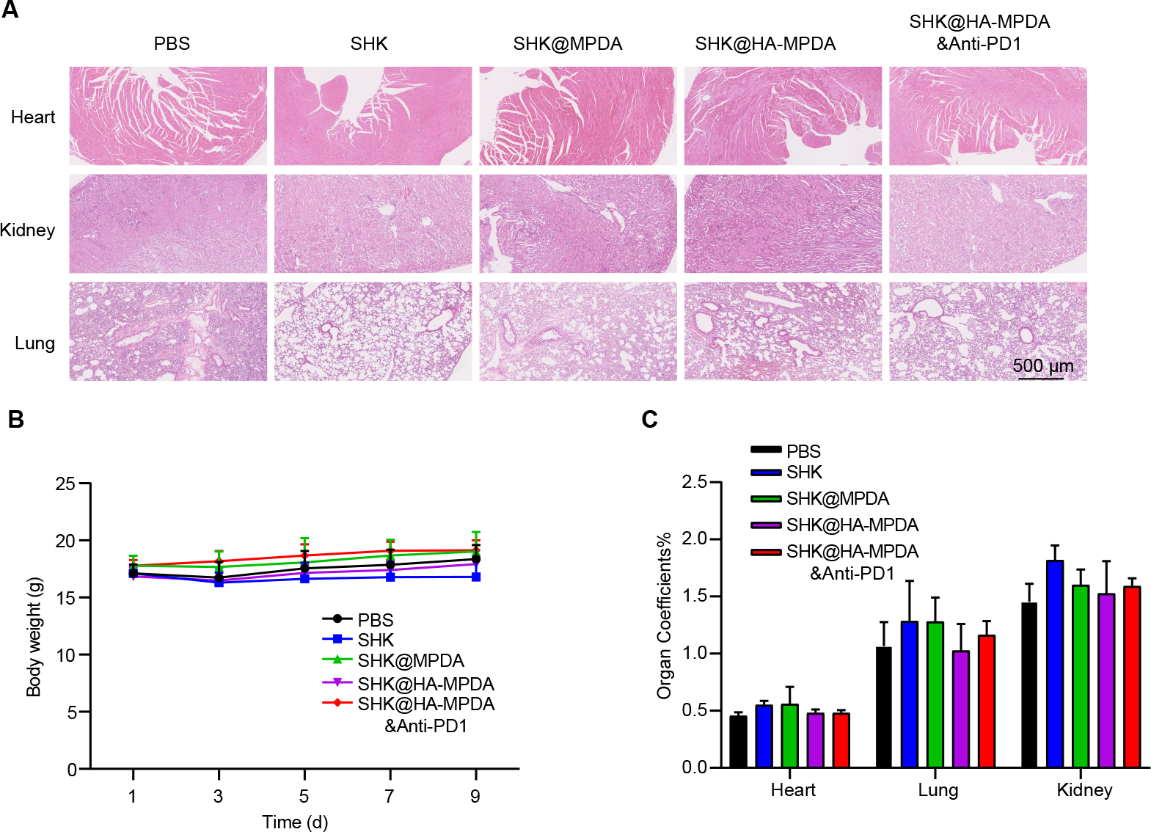

Supplement: Supplementary file 2 — Supplementary Material 2: Fig. S1. The confocal images of CT26 cells and NCM460 cells. Fig. S2. Preliminary evaluation regulating EMT of SHK in a CRLM model. (A) and (B) The expression and grayscale analysis of E-cadherin of epithelial or mesenchymal markers in CT26 and NCM460 cells. (C) Expression of epithelial or mesenchymal markers in CT26 cells after treatment with free SHK. (D) The grayscale analysis of (C). (E) Therapeutic schedule. (F) The expression of E-cadherin of liver tissues in normal and tumor mice. (G) and (H) Expression and grayscale analysis of epithelial or mesenchymal markers of normal or tumor mice. (I) and (J) Western blot analyzed free SHK-regulated EMT in a CRLM model and grayscale analysis of EMT-related makers. (K) Grayscale analysis of Fig. 6G. (L) Grayscale analysis of Fig. 6H. Fig. S3. Flow cytometry gate diagram of CTLs (A) and MDSCs (B) in vitro. Fig. S4. LA inducing EMT depending on TGF/EGF in an EMT model of SW480 cells. (A) and (B) Expression and grayscale analysis of E-cadherin in colorectal cancer cells. (C) and (D) Western blot analysis of epithelial or mesenchymal markers in mesenchymal-like cells with or without TGFβ (10 ng/mL) and SB431542 (5 µM). (E) The LA production of mesenchymal-like cells and epithelial-like cells (TGFβ: 10 ng/mL). (F) and Western blot analysis of LA depended on pH-stimulated TGF/EGF inducing EMT in SW480 cells (LA: 20 mM; SB431542: 5 µM). (G) Grayscale analysis of (F). Fig. S5. The profiles of SHK@MPDA@HA nanosystem. (A–D) The photothermal performance of MPDA in vitro. The temperature changes curve with 808 NIR irradiation of CT26 subcutaneous tumor after treatment with SHK@MPDA (E) and SHK@MPDA@HA (F). The temperature changes curve (G) and image (H) of CT26 subcutaneous tumor with 808 NIR irradiation at 30 h. Fig. S6. Reversion of EMT and remodeling tumor immunodepression microenvironment in early liver metastasis of colorectal cancer. (A) Western blot analysis of epithelial or mesenchymal markers of li [file 13046_2023_2688_MOESM2_ESM.docx]
